# Stiff matrix drives microglial cell migration through Piezo1/Ca^2+^/AKT/cofilin signaling axis-regulated F-actin reassembly

**DOI:** 10.1093/rb/rbaf124

**Published:** 2026-01-12

**Authors:** Xinlan Chen, Zhongchen Li, Junqi Men, Hui Shao, Juncheng Bai, Yingying Guo, Xing Chen, Yubo Fan, Lin-Hua Jiang, Xiaoling Jia

**Affiliations:** Key Laboratory for Biomechanics and Mechanobiology of Ministry of Education, Beijing Advanced Innovation Center for Biomedical Engineering, School of Biological Science and Medical Engineering, and with the School of Engineering Medicine, Beihang University, Beijing 100191, China; Department of Neurosurgery, Liaocheng People’s Hospital, Liaocheng 252000, China; Key Laboratory for Biomechanics and Mechanobiology of Ministry of Education, Beijing Advanced Innovation Center for Biomedical Engineering, School of Biological Science and Medical Engineering, and with the School of Engineering Medicine, Beihang University, Beijing 100191, China; Key Laboratory for Biomechanics and Mechanobiology of Ministry of Education, Beijing Advanced Innovation Center for Biomedical Engineering, School of Biological Science and Medical Engineering, and with the School of Engineering Medicine, Beihang University, Beijing 100191, China; Key Laboratory for Biomechanics and Mechanobiology of Ministry of Education, Beijing Advanced Innovation Center for Biomedical Engineering, School of Biological Science and Medical Engineering, and with the School of Engineering Medicine, Beihang University, Beijing 100191, China; Key Laboratory for Biomechanics and Mechanobiology of Ministry of Education, Beijing Advanced Innovation Center for Biomedical Engineering, School of Biological Science and Medical Engineering, and with the School of Engineering Medicine, Beihang University, Beijing 100191, China; Key Laboratory for Biomechanics and Mechanobiology of Ministry of Education, Beijing Advanced Innovation Center for Biomedical Engineering, School of Biological Science and Medical Engineering, and with the School of Engineering Medicine, Beihang University, Beijing 100191, China; Key Laboratory for Biomechanics and Mechanobiology of Ministry of Education, Beijing Advanced Innovation Center for Biomedical Engineering, School of Biological Science and Medical Engineering, and with the School of Engineering Medicine, Beihang University, Beijing 100191, China; Department of Physiology and Pathophysiology and Sino-UK Joint Laboratory of Brain Function and Injury of Henan Province, Henan Key Laboratory of Neurorestoratology and Protein Modification, The First Affiliated Hospital, Henan Medical University, Xinxiang 453003, China; INSERM U1327 ISCHEMIA 'Membrane Signalling and Inflammation in Reperfusion Injuries’, Faculty of Medicine, University of Tours, Tours 37032, France; School of Biomedical Sciences, Faculty of Biological Sciences, University of Leeds, Leeds LS29JT, UK; Key Laboratory for Biomechanics and Mechanobiology of Ministry of Education, Beijing Advanced Innovation Center for Biomedical Engineering, School of Biological Science and Medical Engineering, and with the School of Engineering Medicine, Beihang University, Beijing 100191, China

**Keywords:** substrate stiffness, Piezo1 channel, microglial cells, migration, F-actin reassembly

## Abstract

The local stiffness of brain tissues increases in pathological states, and then, drives microglial cells to migrate towards stiffer areas for their functioning, but the underlying mechanism is still unclear. Herein, we investigated the role and mechanism of the mechanosensitive Piezo1 channel in matrix stiffness regulation of microglial cell migration using BV2 microglial cell line and mouse primary microglial cells growing on soft (500 Pa) and stiff (20 kPa) polyacrylamide (PA) hydrogels. Compared with soft substrates, stiff substrates promoted cell migration, upregulated the Piezo1 expression, raised intracellular Ca^2+^ concentration ([Ca^2+^]_i_) and favored F-actin reassembly. Cell migration and F-actin assembly were suppressed by inhibiting the Piezo1 channel, reducing the [Ca^2+^]_i_ or inhibiting PI3K/AKT. In addition, stiff substrates induced AKT activation that was reversed by blocking the Piezo1 channel or preventing an increase in [Ca^2+^]_i_. Finally, stiff substrates induced a Piezo1-, Ca^2+^- and AKT-dependent decrease in the phosphorylation level of cofilin, which promotes cofilin severing F-actin, increases G-actin levels and further enhances F-actin reassembly, thereby promoting microglial cell migration. Collectively, our study has revealed that stiff matrix regulates drives microglial cell migration through the Piezo1/Ca^2+^/AKT/cofilin signaling axis-regulated F-actin reassembly. Our findings provide new insights into the mechanisms underlying tissue stiffness regulation of microglial cells occurring in neurological disorders.

## Graphical abstract

**Figure:**
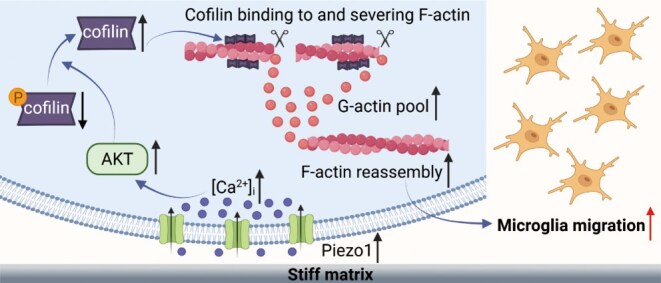

## Introduction

Microglial cells are the immune cells resident in the central nervous system (CNS), actively participating in immune response, maintaining brain homeostasis and promoting injury recovery [1–3]. Under physiological conditions, microglial cells continuously survey brain tissues with minimal displacements [4–6]. In response to pathological stimuli such as infection, damage or neurological disorders, microglial cells become activated, migrate toward the infection/damage sites and phagocytose pathogens or cellular debris, thereby protecting the CNS [7–10]. In addition to biochemical cues, mechanical cues, particularly tissue stiffness, can profoundly influence microglial functions [11–13]. The stiffness of healthy brain tissues is less than 1 kPa [14, 15]. In cases of aging, gliomas, neural implants and neurodegenerative diseases such as Alzheimer’s disease, the local tissue stiffness significantly increases [16, 17]. Accumulating evidence indicates that microglial cells can be activated by stiffness exceeding physiological ranges, leading to changes in morphology, inflammatory responses, phagocytic activity and migration capabilities [8, 18–24]. Despite their critical roles, the regulatory effects of brain tissue stiffness on microglial cell functions such as migration and the underlying molecular mechanisms remain largely unexplored.

Cells sense and transmit mechanical cues into chemical signals via mechanosensors, further influencing cellular functions [25–27]. Piezo1, a mechanosensitive ion channel, has recently garnered increasing attention due to its role in mechanotransduction in the CNS and stiffness-associated alterations of microglial cell function in neurodegenerative diseases [22, 28, 29]. Numerous studies indicate that Piezo1 channel is engaged in the regulation of tissue substrate stiffness affecting microglial cell migration. The activation, blockade or deficiency of Piezo1 has been shown to impact microglial cell migration toward stiff Aβ plaques in the brains of AD mouse models *in vivo* or on substrates with varying stiffness *in vitro* [22, 23, 30, 31]. However, the underlying molecular mechanisms remain unclear.

In general, cells on stiff substrates reorganize their actin cytoskeleton to spread or move [32–34]. Specifically, cytoskeleton remodeling through dynamic actin polymerization and depolymerization significantly impacts cell migration or motility [35–37]. Several recent studies demonstrate that microglial cells migrate in a stiffness-dependent manner, accompanied by actin remodeling, where actin is mostly located in the cell periphery on soft substrates but organized into long stress fibers and distributed throughout cells on stiff substrates [21, 30]. Dynamic actin polymerization and depolymerization, a cyclic process involving globular actin (G-actin) binding to and detaching from the ends of filamentous actin (F-actin) filaments, is tightly regulated by various actin-binding proteins (ABPs) [38, 39]. Cofilin, an actin-depolymerizing factor and a small ABP, severs F-actin filaments and participates in microglial cell migration. Cofilin is inactivated by phosphorylation at Ser-3 and reactivated via dephosphorylation, which enables cofilin to bind to and sever F-actin and thereby increase actin dynamics and F-actin reassembly [40, 41]. Specifically, both actin polymerization and cofilin activation (dephosphorylation) are regulated by intracellular Ca^2+^ [41]. As a Ca^2+^-permeable channel, Piezo1 mainly functions through mediating extracellular Ca^2+^ flux to increase [Ca^2+^]_i_ [42]. Therefore, it is reasonable to hypothesize that Piezo1 is engaged in substrate stiffness-induced regulation of microglial cell migration through Ca^2+^-dependent regulation of cofilin and actin dynamics.

In the present study, the mouse microglial BV2 cell line and primary microglial cells were cultured on soft and stiff polyacrylamide (PA) hydrogels, the mechanical stiffness of which mimics that of healthy and pathological brain tissues, respectively. Combined with RNA-sequencing analysis, we have revealed the role of the Piezo1 channel in raising [Ca^2+^]_i_ to induce PI3K/AKT activation, cofilin phosphorylation and actin cytoskeletal reorganization, thus, mediating stiffness-dependent regulation of microglial cell migration. This study provides novel mechanistic insights into how microglial cells are regulated by disease-associated changes in brain tissue stiffness.

## Materials and methods

### Cell culture

BV2 microglial cells were obtained from the National Infrastructure of Cell Line Resource (Beijing, China) and cultured in high-glucose Dulbecco’s Modified Eagle’s Medium (DMEM, Gibco, 12100046, USA), supplemented with 10% fetal bovine serum (FBS, Procell, 16421050, China), 100 U/mL penicillin and 100 μg/ml streptomycin (Amresco, s0382, s0339, USA). Cells were cultured at 37°C in a humidified incubator with 5% CO_2_. Primary microglial cells were isolated from postnatal C57BL/6J mice aged 0–24 h. Following decapitation, the cortices free of meninges and blood vessels were dissected from the brains in Hanks’ Balanced Salt Solution (HBSS). Tissues were subsequently digested with a 0.25% trypsin-EDTA solution (Gibco, 25200056, USA) at 37°C for 15 min. Cells were filtered through a 40-μm cell strainer and centrifuged at 200 × *g* for 5 min before being resuspended in DMEM/F12 (Gibco, 11320033, USA) supplemented with 10% FBS (Gibco, A5669701, USA) and incubated at 37°C in a humidified atmosphere containing 5% CO_2_. Media were replaced after two days. Microglial cells were isolated from cultures aged 9–11 days by orbital shaking at 200 rpm for 2 h, followed by collection and centrifugation of the culture medium. The purity of microglial cell cultures was validated to be over 99% using immunostaining with an Iba1 antibody (1:1000, Abcam, ab178846, UK). All animal experiments were approved by the Animal Research Ethics Committee of Beihang University.

### Polyacrylamide gel fabrication

PA gels with stiffness less than 1 kPa were often used to simulate healthy brain tissues, while stiffness values of 8–40 kPa were used for pathological tissues [19, 20, 22, 23]. Therefore, in our initial experiments, we used 500 Pa, 1 kPa and 20 kPa to broadly mimic the healthy and pathological brain tissues. PA gels were synthesized according to established protocols [43], and the formulations are presented in Supplementary Table S1. Hydrogel solutions were prepared and then mixed with ammonium persulfate and N, N, N′,N′-tetramethylethylenediamine. Glass coverslips were functionalized with 0.1% NaOH followed by 3-aminopropyltriethoxysilane for hydrophilicity. Hydrogel solutions were pipetted onto a dichlorodimethylsilane-treated hydrophobic coverslips, and hydrophilic coverslips were placed over the droplets to generate flat substrates. A solution of sulfo-succinimidyl-6-(4-azido-2-nitrophenylamino) hexanoate (0.2 mg/mL) was applied to the surface of the hydrogel substrates and subsequently exposed to ultraviolet light (365 nm) for 20 min. The gels were then rinsed with 50 mM HEPES buffer (pH 8.5), incubated overnight at 37°C with 0.1 mg/mL collagen I and washed with PBS prior to cell seeding.

### RNA sequencing

Primary mouse microglial cells were seeded at a density of 1 × 10^6^ cells/well on PA hydrogel substrates with stiffnesses of 500 Pa and 20 kPa. After a 48-h culture, total RNA was extracted using TRIzol™ reagent (Invitrogen, 15596018CN, USA) according to the manufacturer’s protocol, and mRNA was enriched from the total RNA using oligo(dT)-attached magnetic beads. The poly(A) mRNA was fragmented and reverse-transcribed into double-stranded cDNA using random N6 primers. Subsequently, the cDNA underwent end repair, 5′-phosphorylated and had a blunt end with a protruding ‘A’ and a bubble-shaped adapter with a protruding ‘T’ at the 3′ end. The ligation products were amplified via PCR using specific primers, and single-stranded circular DNA libraries were subsequently constructed from the amplified products using a bridged primer. The libraries were subjected to quality assessment prior to sequencing. Sequencing was based on DNBSEQ platform provided by BGI (Beijing Genomics Institute, BGI, China). The resulting sequencing data underwent quality control (QC) to ensure suitability for downstream analysis. Clean reads were aligned to reference sequences according to BGI protocols, followed by a secondary QC step to evaluate alignment rate and their distribution. Gene expression quantification and differential analysis were subsequently conducted. Differentially expressed genes (DEGs) were analyzed for Gene Ontology (GO) enrichment and Kyoto Encyclopedia of Genes and Genomes (KEGG) pathways enrichment, with significant functional terms identified at a P-value threshold of < 0.05.

### Western blotting

BV2 cells were lysed using RIPA lysis buffer (NCM Biotech, China) supplemented with phenylmethylsulfonyl fluoride (Beyotime, P1005, China) to inhibit protease activity. The lysate was incubated at 4°C for 30 min and clarified by centrifugation at 12 000 rpm for 10 min. Protein concentrations were determined using the Pierce BCA Protein Assay Kit (Thermo Fisher, 23227, USA). Proteins were denatured in loading buffer at 100°C for 5 min and 20 μg of total protein was separated by 10% SDS-PAGE (sodium dodecyl sulfate-polyacrylamide gel electrophoresis) gels prior to transfer onto polyvinylidene fluoride membranes (Merck, IPVH00010, Germany). Membranes were blocked with 5% bovine serum albumin (BSA) in Tris-buffered saline containing 0.1% Tween-20 for 1 h at room temperature. Primary antibodies, including anti-Piezo1 (1:1000, Proteintech, 28511-1-AP, USA), anti-AKT (1:2000, Cell Signaling Technology, 4691, USA), anti-p-AKT (1:2000, Cell Signaling Technology, 4060, USA), anti-cofilin (1:200, Santa Cruz, sc376476, USA), anti-p-cofilin (1:200, Santa Cruz, sc365882, USA) and anti-GAPDH (1:500, Goodhere, AB-P-R001, China), were incubated overnight at 4°C. Horseradish peroxidase-conjugated secondary antibodies, including goat anti-rabbit IgG (1:2000, ZSGB-BIO, ZB2301, China) and goat anti-mouse IgG (1:2000, ZSGB-BIO, ZB2305, China), were used for immunodetection. Protein signals were visualized using an enhanced chemiluminescence substrate (Allmeek, AM6601, China) and quantified by ImageJ software.

### Calcium imaging

Fluo-4 acetoxymethyl ester (AM) (Thermo Fisher, F14201, USA) was used to monitor changes in the [Ca^2+^]_i_. Cells were seeded on PA hydrogel substrates at a density of 2 × 10^4^ cells/cm^2^ and cultured under standard conditions. After washing with HBSS buffer, cells were incubated with 5 μM Fluo-4 AM for 30 min at 37°C. Following incubation, cells were washed twice with D-Hank’s solution and maintained in D-Hank’s medium prior to subsequent treatments. Fluo-4 fluorescence intensity was recorded at intervals of 500 ms over a 5-min period using a Dragonfly confocal microscope equipped with a live-cell imaging module. To investigate the role of the Piezo1 channel in mediating Ca^2+^ influx, 2 mM CaCl_2_ and 1 μM GsMTx4 (Alomone Labs, STG-100, Israel) were applied during image acquisition, as indicated in the figures. Changes in Fluo-4 fluorescence intensity were analyzed using ImageJ software [44, 45].

### qRT-PCR

Total RNA was extracted from primary microglial cells using TRIzol reagent, followed by reverse transcription into cDNA using Evo M-MLV RT Premix (Accurate Biology, AG11706, China). Gene expression levels of target transcripts were determined by quantitative real-time PCR (qPCR) on a QuantStudio™ 3 Real-Time PCR System. The cDNA was mixed with SYBR Green Premix Kit (Accurate Biology, AG11739, China) and gene-specific primers, then amplified using a thermal cycling program consisting of an initial denaturation at 95°C for 30 s, followed by 40 cycles of denaturation at 95°C for 10 s and annealing at 56°C for 34 s. The primer sequences used were as follows: Piezo1, 5′-AGGACTTCCCCACCTATTGG-3′ (forward) and 5′-CCAGGGATGAGGATACTGGAAAA-3′ (reverse); AKT1, 5′-CGCTACTATGCCATGAAGATCCT-3′ (forward) and 5′-GAATGAGTACTTGAGGGCCGTAA-3′ (reverse); Pik3ca, 5′-CACTGTGGTTGAATTGGGAGAAC-3′ (forward) and 5′-TGCATGATGGTGTGAGAGTTTCT-3′ (reverse); GAPDH, 5′-GTTTGTGATGGGTGTGAACC-3′ (forward) and 5′-TCTTCTGAGTGGCAGTGATG-3′ (reverse). mRNA expression levels were normalized to GAPDH and calculated using the 2^-△△Ct^ method [46].

### Transfection with siRNA

Primary microglial cells were seeded at a density of 2 × 10^4^ cells/cm^2^ and cultured until cell confluence reached approximately 40%. Thereafter, the cells were transfected with Piezo1-specific siRNA (5′-GUCUCAAGAACUUCGUAGATT-3′) and negative control siRNA (5′-UUCUCCGAACGUGUCACGUTT-3′) using RFect^PM^ (BAIDAI, 10014, China). The cells were replated 72 h post-transfection onto PA hydrogel substrates for experimentation.

### Live-cell imaging

BV2 cells and primary microglial cells were seeded onto PA hydrogel substrates at a density of 1 × 10^4^ cells/cm^2^ overnight and treated with 5 μM Yoda1 (AbMole, M9372, China) for 1 h. Cells were treated with 1 μM GsMTx4, 10 μM BAPTA-AM (AbMole, M4973, China), 20 μM LY294002 (AbMole, M1925, China) or 10 μM U0126 (AbMole, M1977, China) for 1 h prior to tracking cell migration using a Leica SP8X scanning confocal microscope equipped with a live-cell module. Autofocus measurements were recorded at 10-min intervals and the recording lasted a total duration of 8 h. Cell trajectories and traveling distances were quantified using ImageJ software.

### Immunofluorescent staining

BV2 cells and primary microglial cells were cultured on PA hydrogel substrates, washed with PBS and fixed with 4% paraformaldehyde for 20 min at room temperature. The cells were permeabilized with 0.2% Triton X-100 for 7 min followed by blocking with 5% BSA for 1 h. Primary antibodies were diluted in PBS containing 1% BSA and applied to the samples, which were then incubated overnight at 4°C. The primary antibodies and dilutions were as follows: Piezo1 (1:1000, Proteintech, 28511-1-AP, USA), AKT (1:400, Cell signaling Technology, 4691, USA) and cofilin (1:200, Santa Cruz, sc376476, USA). Secondary antibodies, F-actin antibody and G-actin antibody were diluted in PBS and incubated at room temperature for 1 h. The secondary detection reagents included: YF^®^ 488-conjugated goat anti-rabbit IgG (1:200, UElandy, Y6105S, China), YF^®^ 647-conjugated goat anti-mouse IgG (1:200, UElandy, Y6108S, China), Alexa Fluor™ 488-conjugated DNase I (1:200, Thermo Fisher, D12371, USA) and Alexa Fluor™ 555 Phalloidin (1:200, Thermo Fisher, A23055, USA). DAPI (1:1000, Merck, D9542, Germany) was used to counterstain the nuclei. Fluorescence images were captured using a Dragonfly confocal laser scanning microscope, and fluorescence intensity was analyzed using ImageJ software.

### Data presentation and statistical analysis

Data are presented as mean ± standard deviation (SD) where appropriate. A two-tailed Student’s *t*-test was employed to determine the significance of differences between two groups. For comparisons among multiple groups, one-way analysis of variance (ANOVA) was performed, followed by Fisher’s least significant difference (LSD) *post hoc* test. *P *< 0.05 was considered statistically significant.

## Results

### Piezo1 is upregulated in microglial cells and involved in stiff substrate-induced cell migration

**Figure 1:**
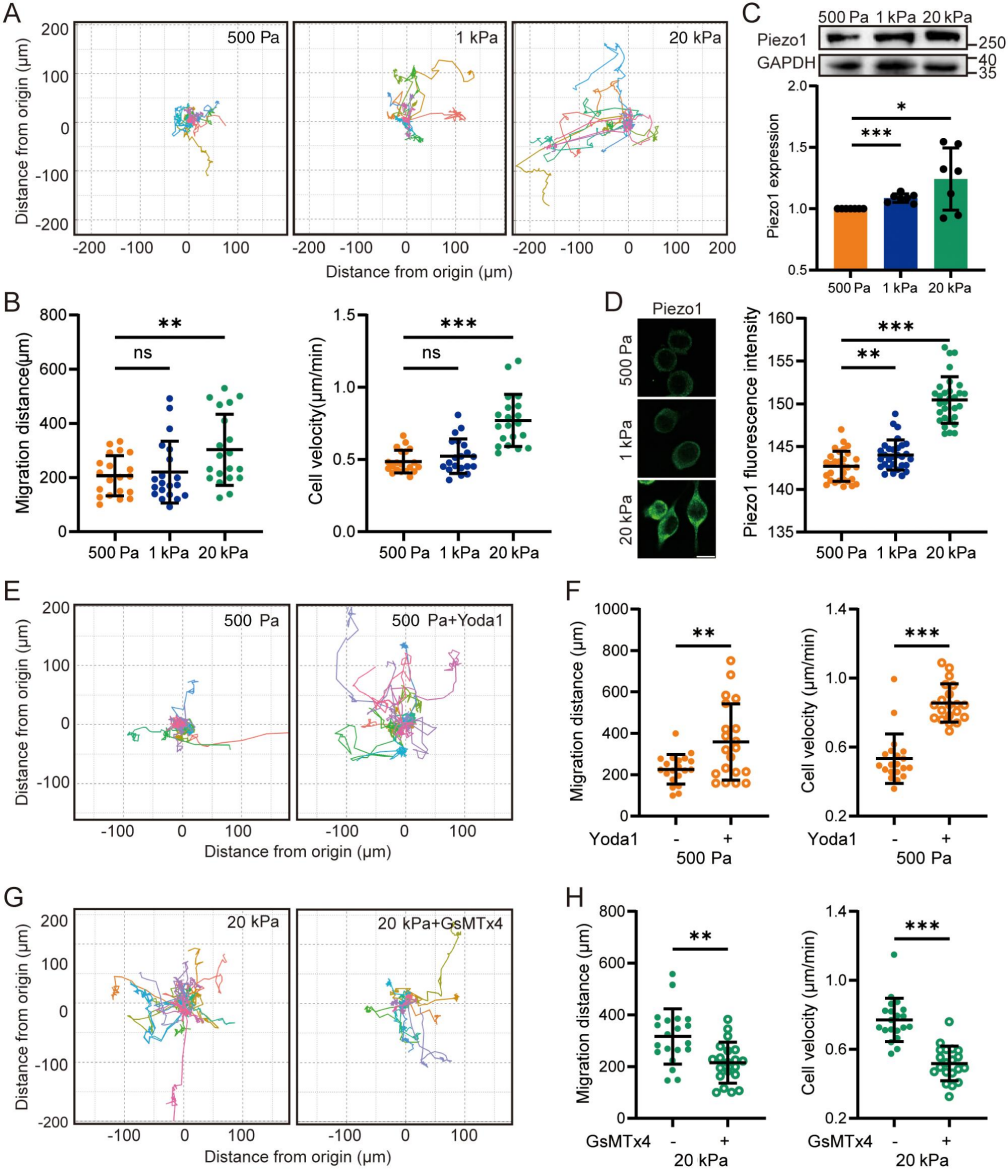
The Piezo1 channel is involved in stiff substrate-induced BV2 cell migration. (**A**, **B**) Cell migration over an 8-h period cultured on substrates of 500 Pa, 1 kPa and 20 kPa. (**A**) Migration trajectories of individual cells; (**B**) Quantitative analysis of total migration distance (left) and average velocity (right) (*n* = 20 cells). (**C**) Representative Western blots showing (top) and quantification (bottom) of the Piezo1 protein expression in cells on 500 Pa, 1 kPa and 20 kPa substrates (*n* = 7 repeats). (**D**) Representative immunofluorescence images showing the Piezo1 protein expression (left) and quantification of fluorescence intensity (right) for cells on 500 Pa, 1 kPa and 20 kPa substrates (scale bar: 10 µm; *n* = 30 cells). (**E**, **F**) Cell migration on soft substrates (500 Pa), which were pretreated with DMSO or 5 μM Yoda1 for 1 h and subsequently tracked for 8 h. (**E**) Migration trajectories of individual cells; (**F**) Quantitative analysis of total migration distance (left) and average velocity (right) (*n* = 20 cells). (**G**, **H**) Cell migration on stiff substrates (20 kPa) that were pretreated with DMSO or 1 μM GsMTx4 for 1 h and subsequently tracked for 8 h. (**G**) Migration trajectories of individual cells; (**H**) Quantitative analysis of total migration distance (left), and average velocity (right) (*n* = 20 cells). ns, not significant; *P < 0.05; **P < 0.01; ***P < 0.001.

The CNS tissues are among the softest tissues in the body, with physiological stiffness ranging from 100 to 1000 Pa [47]. Under pathological conditions such as AD, brain tissue stiffness increases due to Aβ deposition and varies according to the stage of disease progression and the specific location of the affected regions. The areas with Aβ deposition are characterized by a rigid core and a softer periphery. Reportedly, PA gels with a stiffness range of 8–40 kPa are used to simulate pathological mechanical environments [19, 20, 22, 23]. To broadly mimic the extracellular matrix microenvironments of microglial cells, we established three distinct substrate stiffness levels: 500 Pa to represent physiological stiffness, 1 kPa as the transition point between physiological and pathological stiffness, and 20 kPa to reflect pathological stiffness. We first assessed the migration of BV2 microglial cells using live-cell imaging to monitor cellular movement on substrates and reconstructed their migration trajectories (Figure 1A). The results demonstrated that the migratory activity of BV2 cells was significantly higher on 20 kPa substrates compared to those with 500 Pa or 1 kPa stiffness, as evidenced by increased migration distance and velocity (Figure 1B). To investigate the potential role of the Piezo1 channel in mechanosensing and cell migration, we analyzed Piezo1 expression by Western blotting. Compared to cells cultured on 500 Pa substrates, Piezo1 protein expression in BV2 cells was moderately elevated on 1 kPa substrates and markedly upregulated on 20 kPa substrates (Figure 1C). Immunofluorescence staining further confirmed the upregulation of Piezo1 on stiff substrates (Figure 1D). Based on these findings, substrates with stiffnesses of 500 Pa and 20 kPa were selected to mimic the soft and stiff extracellular matrices of brain tissue under physiological and pathological conditions, respectively, to investigate the underlying mechanisms driving microglial cell migration.

To investigate the role of the Piezo1 channel in substrate stiffness-mediated regulation of cell migration, we examined the effects of activating or inhibiting Piezo1 on the migration of BV2 microglial cells. Treatment of cells cultured on soft substrates with Yoda1, a potent and selective Piezo1 agonist, significantly enhanced cell migration (Figure 1E and F). Conversely, treatment of cells on stiff substrates with GsMTx4, an inhibitor of Piezo1, significantly suppressed migration (Figure 1G and H). Consistently, Piezo1 knockdown had an inhibitory effect on BV2 cell migration on stiff substrates (Supplementary Figure S1A and B). Collectively, these results indicate that the Piezo1 channel mediates the substrate stiffness regulation of microglial cell migration.

### Piezo1-mediated rise in [Ca^2+^]_i_ is essential for stiff substrate-induced cell migration and F-actin remodeling

**Figure 2:**
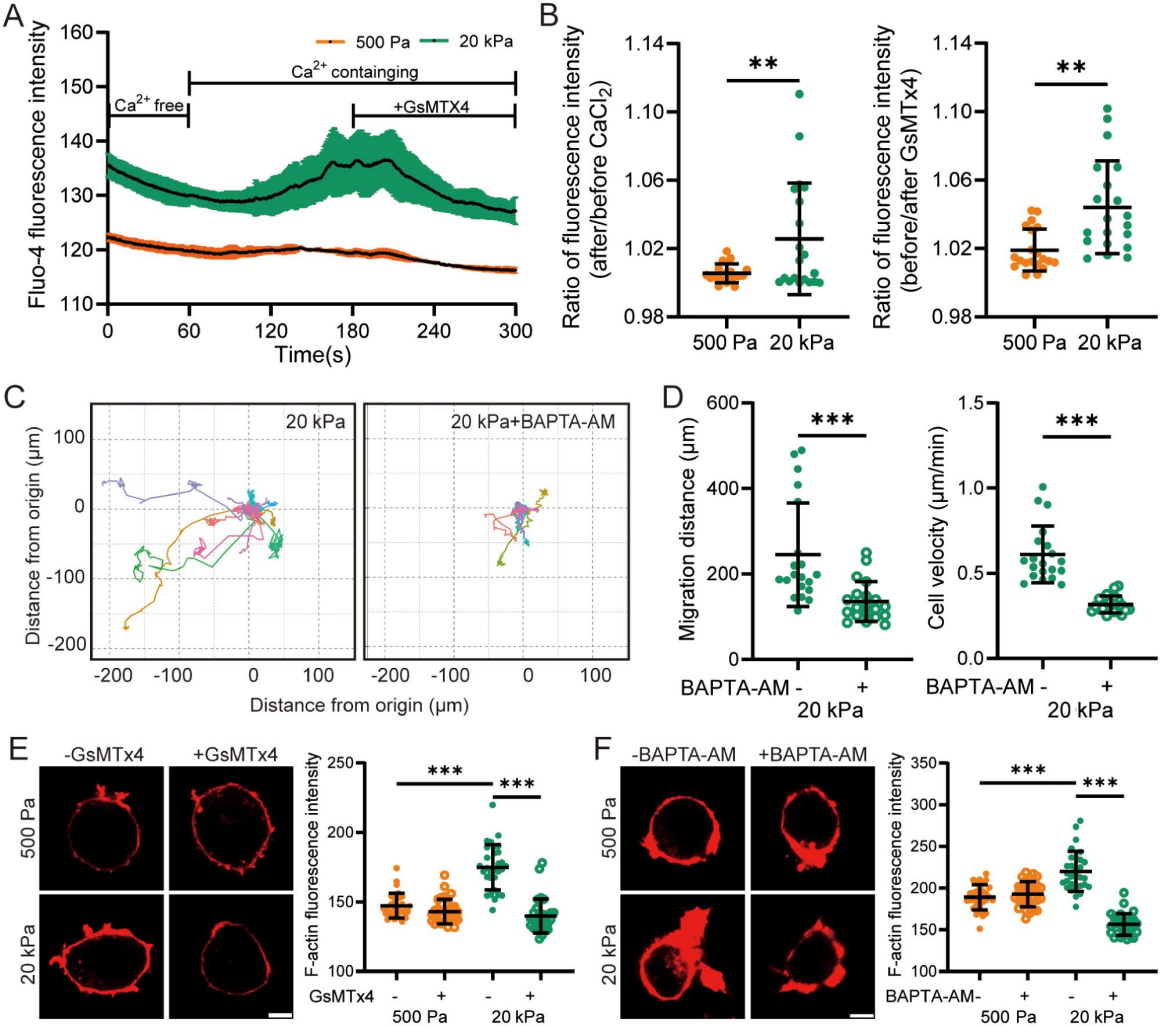
Piezo1-Mediated Ca^2+^ influx promotes migration and F-actin assembly in BV2 cells on stiff substrates. (**A**, **B**) Fluo-4 fluorescence intensity in cells on 500 Pa or 20 kPa substrates in the absence and then presence of Ca^2+^ and 1 μM GsMTx4 over a 5-min period. 2 mM CaCl_2_ was added at 60 s, and GsMTx4 at 180 s as indicated (*n* = 3 cells). (**A**) Temporal changes in fluo-4 fluorescence intensity for individual cells; (**B**) Quantitative analysis of fluo-4 fluorescence intensity ratios after and before the addition of CaCl_2_ (left), and before and after treatment with GsMTx4 (right) (*n* = 20 cells). (**C**, **D**) Migration of cells seeded on 20 kPa substrates that were pretreated with DMSO or 10 μM BAPTA-AM for 1 h and subsequently tracked for 8 h. (**C**) Migration trajectories of individual cells; (**D**) Quantitative analysis of total migration distance (left) and average velocity (right) (*n* = 20 cells). (**E**, **F**) Representative immunofluorescence images showing F-actin and quantification of fluorescence intensity in cells seeded on 500 Pa and 20 kPa substrates. Cells were pretreated with 1 μM GsMTx4 (**E**) or 10 μM BAPTA-AM (**F**) for 1 h and subsequently cultured for an additional 12 h (scale bar: 5 µm; *n* = 30 cells). **P < 0.01; ***P < 0.001.

Piezo1 senses substrate stiffness to modulate [Ca^2+^]_i_, thereby regulating microglial polarization, chemotaxis and phagocytosis [18, 19]. We next employed Fluo-4 AM as a Ca^2+^ indicator to investigate the dynamic changes in [Ca^2+^]_i_ in microglial cells in response to substrate stiffness. A substantial Ca^2+^ influx was observed upon extracellular Ca^2+^ addition in cells cultured on stiff substrates, whereas minimal Ca^2+^ influx occurred in cells on soft substrates. Furthermore, Ca^2+^ influx in cells on stiff substrates was significantly reduced by treatment with GsMTx4 (Figure 2A and B). Piezo1 knockdown also attenuated stiff substrate-induced increase in [Ca^2+^]_i_ (Supplementary Figure S2A). Cell migration on stiff substrates was also inhibited following pretreatment with BAPTA-AM to chelate intracellular Ca^2+^ and prevent [Ca^2+^]_i_ elevation (Figure 2C and D). These results indicate that the Piezo1-mediated increase in [Ca^2+^]_i_ promotes microglial cell migration induced by stiff substrates.

Cell migration is tightly regulated by cytoskeletal remodeling. The cytoskeleton generates protrusive and contractile forces through dynamic processes of actin disassembly and reassembly, thereby modulating cellular movement [36, 48]. The Piezo1 channel has been shown to play a critical role in regulating actin dynamics in response to mechanical stiffness stimuli [49]. To further elucidate the underlying mechanisms of cell migration, we examined F-actin expression by staining with phalloidin. The results showed that F-actin levels were significantly higher in cells cultured on stiff substrates compared to those on soft substrates. Moreover, F-actin levels in cells on stiff substrates were markedly reduced by treatment with GsMTx4 (Figure 2E) or BAPTA-AM (Figure 2F). These results suggest that the Piezo1-mediated increase in [Ca^2+^]_i_ is crucial for F-actin remodeling and the subsequent migration of microglial cells.

### Piezo1 is essential for promotion of migration of primary microglial cells on stiff substrates

**Figure 3:**
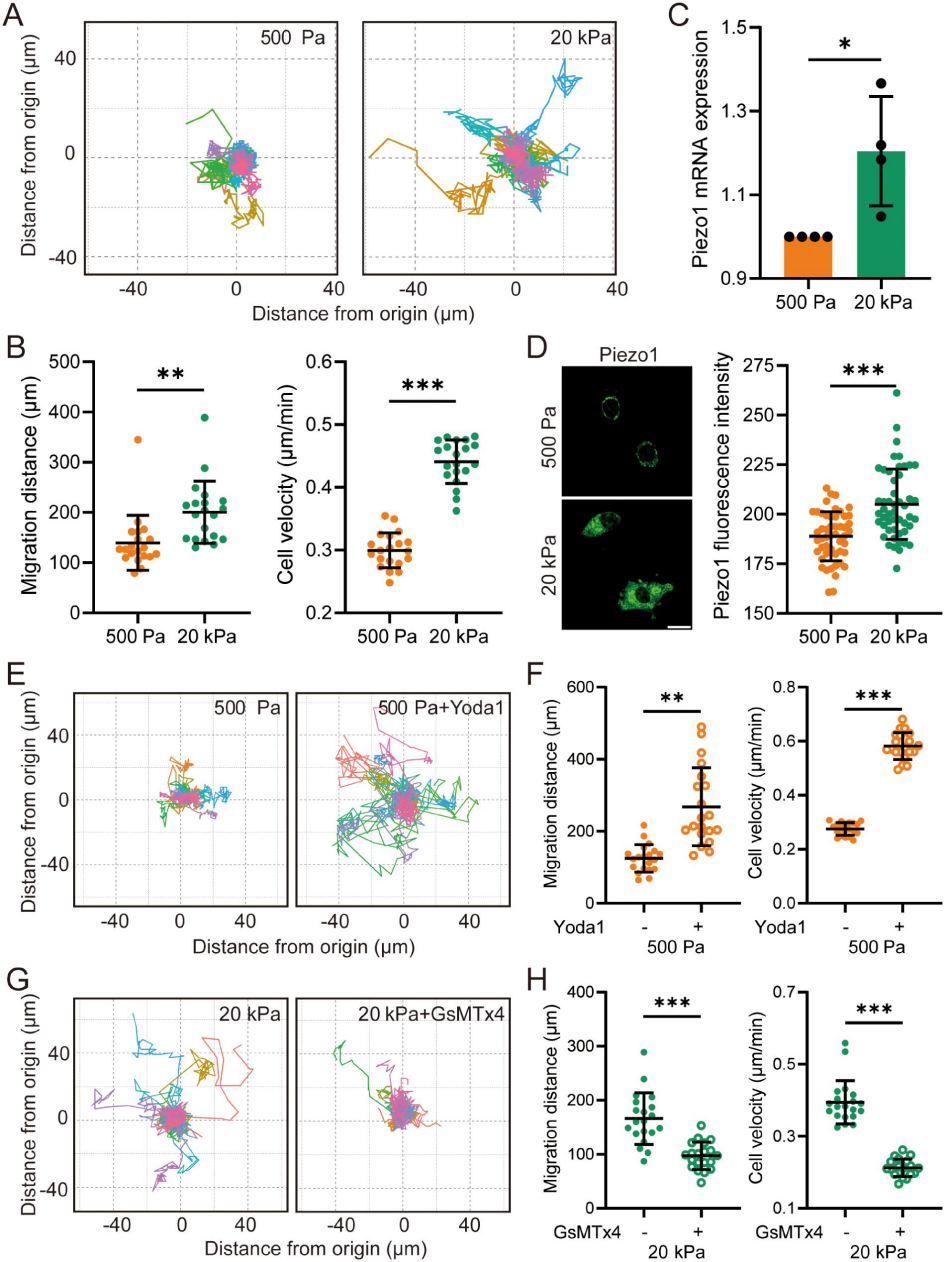
The Piezo1 channel mediates stiff substrate-induced migration of primary microglial cells. (**A**, **B**) Migration analysis of cells over an 8-h period seeded on 500 Pa and 20 kPa substrates. (**A**) Migration trajectories of individual cells; (**B**) Quantitative analysis of total migration distance (left) and average velocity (right) (*n* = 20 cells). (**C**) Quantitative analysis of the Piezo1 mRNA expression in cells seeded on 500 Pa and 20 kPa substrates (*n* = 4 repeats). (**D**) Representative immunofluorescence images showing Piezo1 expression (left) and quantification of fluorescence intensity (right) in cells on 500 Pa and 20 kPa substrates (scale bar: 10 µm, *n* = 30 cells). (**E**, **F**) Migration analysis of cells seeded on soft substrates (500 Pa) pretreated with DMSO or 5 μM Yoda1 for 1 h and subsequently tracked for 8 h. (**E**) Migration trajectories of individual cells; (**F**) Quantitative analysis of total migration distance (left) and average velocity (right) (*n* = 20 cells). (**G**, **H**) Migration analysis of cells on stiff substrates (20 kPa) pretreated with DMSO or 1 μM GsMTx4 for 1 h and subsequently tracked for 8 h (*n* = 20 cells). (**G**) Migration trajectories of individual cells; (**H**) Quantitative analysis of total migration distance (left) and average velocity (right) (*n* = 20 cells). *P < 0.05; **P < 0.01; ***P < 0.001.

BV2 cells are widely used in the study of microglial cell function but do not fully recapitulate all functional characteristics of primary microglia in brain tissue [44–46]. To validate the above findings obtained with BV2 cells, we isolated primary microglial cells from mice and cultured them on substrates of defined stiffness. The results showed that primary microglial cells exhibited enhanced migratory capacity on stiff substrates, as evidenced by increased migration distance and velocity (Figure 3A and B). Piezo1 expression was also upregulated in primary microglial cells cultured on stiff substrates (Figure 3C and D). Furthermore, migration on soft substrates was significantly enhanced by treatment with Yoda1 (Figure 3E and F), whereas migration on stiff substrates was attenuated by GsMTx4 treatment (Figure 3G and H) or Piezo1 knockdown (Supplementary Figure S1C and D). These findings from primary microglial cells are highly consistent with those derived from BV2 cells and collectively provide strong evidence that the Piezo1 channel serves as a critical molecular mediator in sensing substrate stiffness and promoting microglial cell migration.

### Piezo1-mediated rise in [Ca^2+^]_i_ is crucial for stiff substrate-induced migration and F-actin remodeling in primary microglial cells

**Figure 4:**
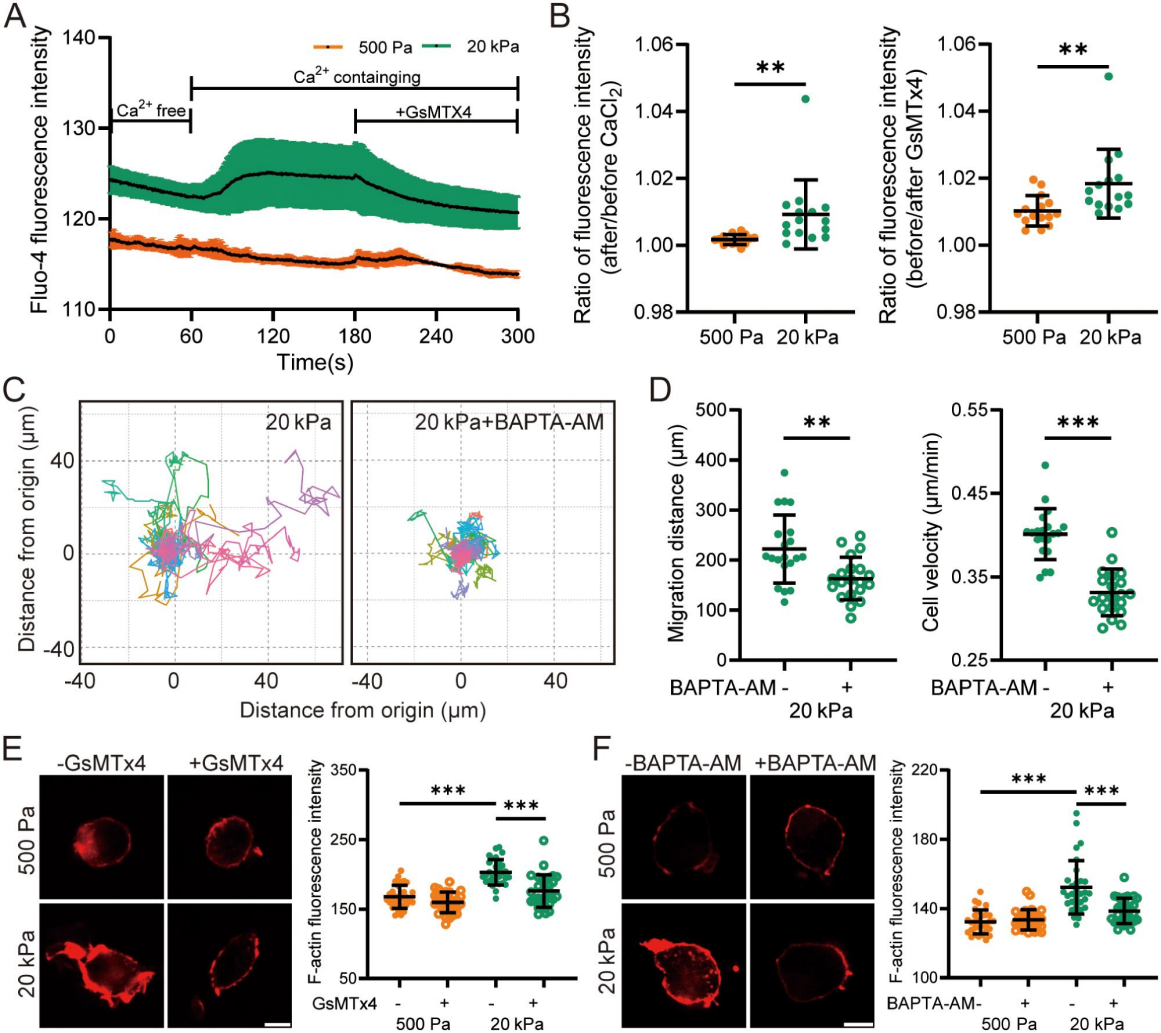
Piezo1-mediated Ca^2+^ influx promotes migration and F-actin reassembly in primary microglial cells on stiff substrates. (**A**, **B**) Fluo-4 fluorescence intensity in BV2 cells on 500 Pa or 20 kPa substrates in the absence and presence of Ca^2+^ and 1 μM GsMTx4 over a 5-min period. 2 mM CaCl_2_ was added at 60 s, and GsMTx4 at 180 s as indicated (*n* = 3 cells). (**A**) Temporal changes in fluo-4 fluorescence intensity for individual cells; (**B**) Quantitative analysis of fluorescence intensity ratios after and before the addition of CaCl_2_ (left), and before and after treatment with GsMTx4 (right) (*n* = 15 cells). (**C**, **D**) Migration of primary microglial cells on 20 kPa substrates pretreated with DMSO or 10 μM BAPTA-AM for 1 h and subsequently tracked for 8 h. (**C**) Migration trajectories of individual cells; (**D**) Quantitative analysis of total migration distance (left) and average velocity (right) (*n* = 20 cells). (**E**, **F**) Representative immunofluorescence images showing F-actin and quantification of fluorescence intensity in primary microglial cells seeded on 500 Pa and 20 kPa substrates. Cells were pretreated with 1 μM GsMTx4 (**E**) or 10 μM BAPTA-AM (**F**) for 1 h and subsequently cultured for an additional 12 h (scale bar: 5 µm; *n* = 30 cells). **P < 0.01; ***P < 0.001.

We next evaluated the role of Piezo1-mediated Ca^2+^ signaling in regulating the migration of primary microglial cells cultured on soft and stiff substrates. An increase in [Ca^2+^]_i_ was detected in primary microglial cells on stiff substrates, which was effectively inhibited by treatment with GsMTx4 (Figure 4A and B). Piezo1 knockdown also reduced stiff substrate-induced increase in [Ca^2+^]_i_ (Supplementary Figure S2B). The migratory capacity of primary microglial cells on stiff substrates was significantly suppressed by pretreatment with BAPTA-AM to chelate intracellular Ca^2+^ (Figure 4C and D). Moreover, F-actin levels were enhanced in primary microglial cells cultured on stiff substrates, and this enhancement was markedly reduced following treatment with GsMTx4 or BAPTA-AM (Figure 4E and F). Taken together, these results provide further evidence that the Piezo1-mediated elevation of [Ca^2+^]_i_ is critical for stiff substrate-induced F-actin remodeling and microglial cell migration.

### Bioinformatic analysis of the signaling pathways involved in substrate stiffness regulation of microglial cell migration

**Figure 5:**
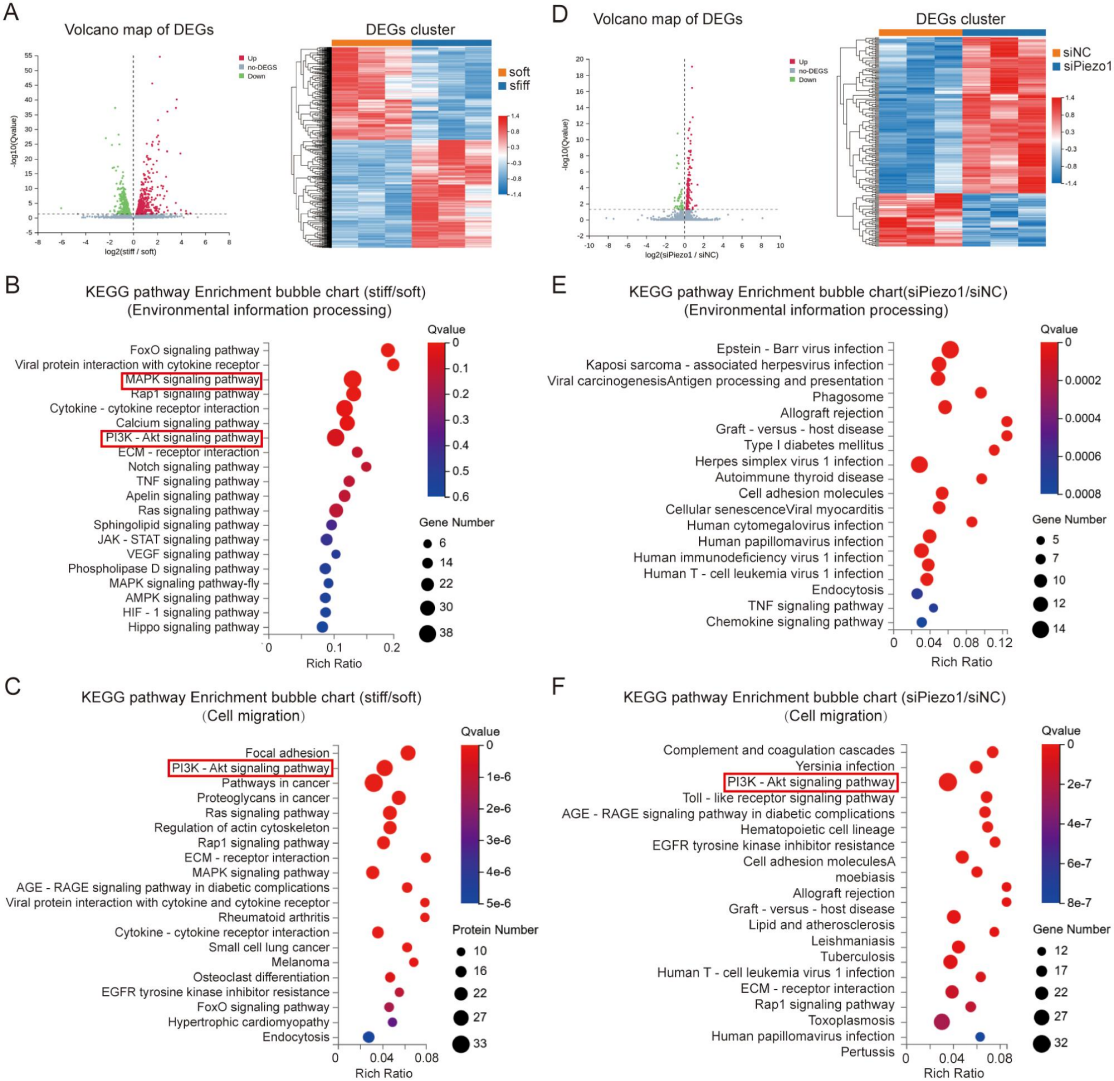
RNA-Seq analysis of the signaling pathways driving substrate stiffness-induced regulation of migration of primary microglial cells. (**A**) Statistical analysis of DEGs in primary microglial cells cultured on 500 Pa (soft) or 20 kPa (stiff) substrates, including a volcano map (left) and cluster heatmaps (right). (**B**) KEGG pathway enrichment analysis of DEGs associated with environmental information processing influenced by substrate stiffness in primary microglial cells. Significant signaling pathways are highlighted by rectangles. (**C**) KEGG enrichment analysis of DEGs involved in cell migration affected by substrate stiffness in primary microglial cells. (**D**) Statistical analysis of DEGs in primary microglial cells following transfection with negative control siRNA (siNC) or Piezo1-specific siRNA (siPiezo1) and culture on stiff substrates, including a volcano plot (left) and cluster heatmaps (right). (**E**) KEGG pathway enrichment analysis of DEGs associated with environmental information processing following Piezo1 knockdown on stiff substrates. (**F**) KEGG pathway enrichment analysis of DEGs involved in cell migration processes affected by silencing Piezo1 expression in primary microglial cells cultured on stiff substrates.

Activation of Piezo1 channels increases [Ca^2+^]_i_, which subsequently activates downstream signaling pathways, thereby facilitating mechanotransduction in various cell types [50–54]. In microglial cells, studies have shown that the JNK, p38 and NF-κB signaling pathways are modulated by chemical activation of the Piezo1 channel [28, 55, 56]. However, the specific downstream signaling pathways through which Piezo1 mediates mechanical regulation of microglial cell function remain incompletely understood. To identify potential signaling mediators, we performed RNA sequencing on primary microglial cells cultured on soft and stiff substrates as described above. The results revealed upregulation of 629 genes and downregulation of 541 genes in cells on stiff substrates compared to those on soft substrates (Figure 5A). KEGG and GO are widely used standardized databases for classifying gene functions via hierarchical frameworks. To identify the pathways mediating microglial responses to substrate stiffness, we performed KEGG pathway enrichment analysis focusing on environmental information processing and found that most differentially expressed genes (DEGs) are enriched in the MAPK and PI3K/AKT signaling pathways (Figure 5B). To link stiffness-responsive pathways to cell migration, we first extracted DEGs annotated to the GO biological process term ‘positive regulation of cell migration’, yielding a set of 43 migration-related DEGs (Supplementary Figure S3). Moreover, KEGG pathway enrichment analysis was performed on these genes, revealing significant enrichment of the PI3K/AKT signaling pathway (Figure 5C). These data suggest that the PI3K/AKT signaling pathway plays a critical role in the substrate stiffness-mediated regulation of microglial cell function. Subsequently, we performed RNA sequencing on primary microglial cells that were pre-transfected with siRNA to reduce Piezo1 expression and cultured on stiff substrates. A total of 151 upregulated and 51 downregulated genes were differentially expressed following genetic knockdown of Piezo1 (Figure 5D). KEGG pathway enrichment analyses were conducted as described above (Figure 5E and F). Notably, the PI3K/AKT signaling pathway remained significantly associated with cell migration (Figure 5F). These results suggest that the PI3K/AKT signaling pathway is a critical component of the molecular mechanism underlying substrate stiffness-mediated regulation of microglial cell migration.

### Piezo1/Ca^2+^-mediated regulation of the AKT signaling and cofilin activation is essential for F-actin reassembly and stiff substrate-induced cell migration

**Figure 6:**
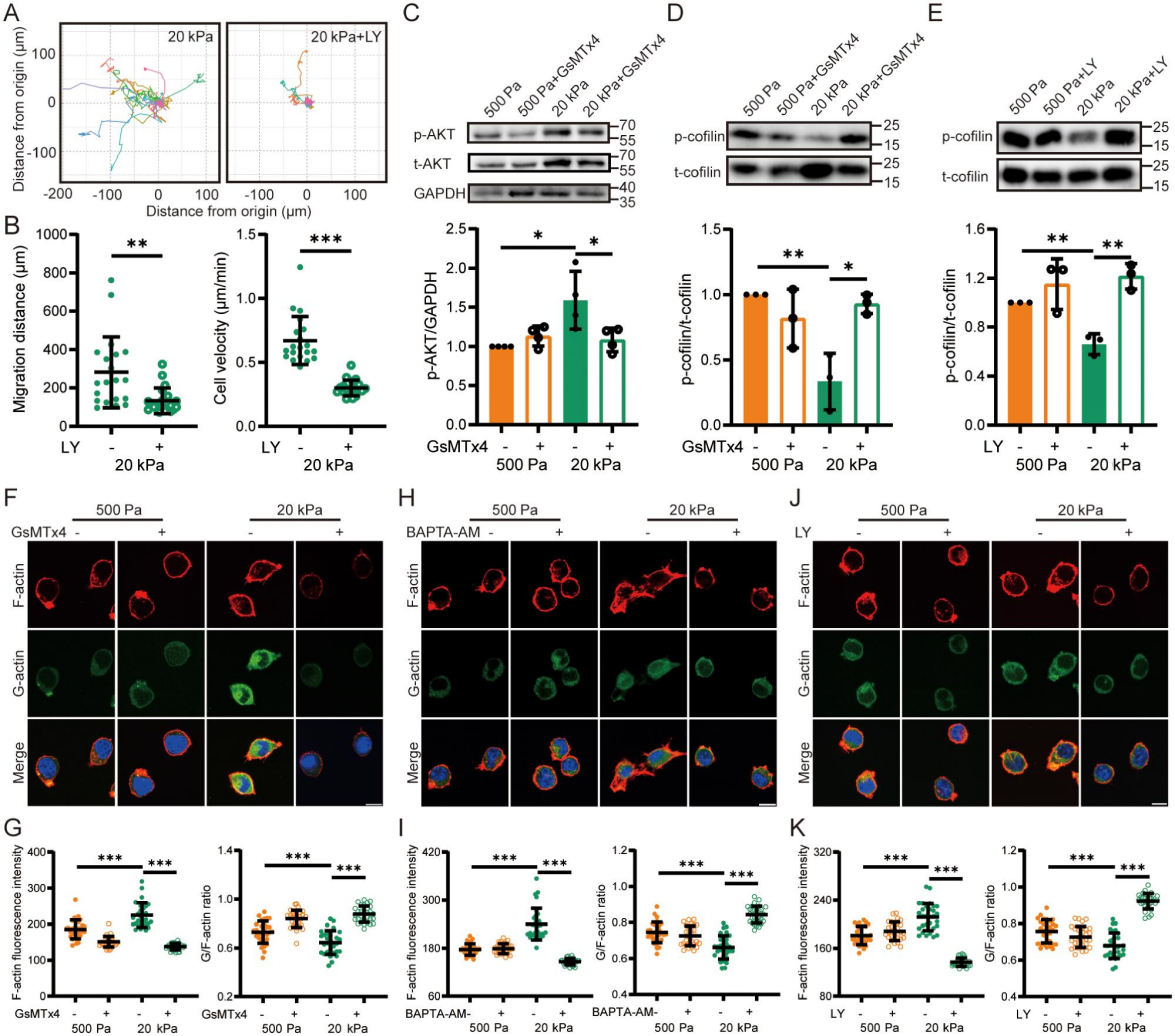
Piezo1-mediated and Ca^2+^-dependent AKT activity is critical for substrate stiffness regulation of cell migration, cofilin phosphorylation and actin dynamics in BV2 cells. (**A**, **B**) Migration analysis of BV2 cells seeded on 20 kPa substrates and pretreated with DMSO or 20 μM LY for 1 h and subsequently tracked for 8 h. (**A**) Migration trajectories of individual cells; (**B**) Quantitative analysis of total migration distance (left) and average velocity (right) (*n* = 20 cells). (**C**) Representative Western blots showing (top) and quantification of p-AKT protein expression (bottom) in cells on 500 Pa and 20 kPa substrates (*n* = 4 repeats). Cells were pretreated with DMSO or 1 μM GsMTx4 for 1 h and subsequently cultured for a further 12 h. (**D**, **E**) Representative Western blots showing phosphorylation of cofilin (p-cofilin) and total cofilin (t-cofilin) expression (top) and quantification of p-cofilin to t-cofilin ratio (bottom) in cells seeded on 500 Pa and 20 kPa substrates (*n* = 3 repeats). Cells were pretreated with 1 μM GsMTx4 (**D**) or 20 μM LY (**E**) for 1 h and subsequently cultured for a further 12 h. (**F**, **G**) Immunofluorescent staining of F-actin and G-actin in cells on 500 Pa and 20 kPa substrates. Cells were pretreated with DMSO or 1 μM GsMTx4 for 1 h and subsequently cultured for a further 12 h. (**F**) Representative images showing expression of F-actin and G-actin; (**G**) Quantification of F-actin protein expression (left) and G-actin/F-actin ratio (right) (*n* = 30 cells). (**H**, **I**) Immunofluorescent staining of F-actin and G-actin in cells seeded on 500 Pa and 20 kPa substrates. Cells were pretreated with DMSO or 10 μM BAPTA-AM for 1 h and subsequently cultured for a further 12 h. (**H**) Representative images showing F-actin and G-actin. (**I**) Quantification of F-actin protein expression (left) and G-actin/F-actin ratio (right) (*n* = 30 cells). (**J**, **K**) Immunofluorescent staining of F-actin and G-actin in cells on 500 Pa and 20 kPa substrates. Cells were pretreated with DMSO or 20 μM LY for 1 h and subsequently cultured for a further 12 h. (**J**) Representative images showing expression of F-actin and G-actin; (**K**) Quantification of F-actin protein expression (left) and G-actin/F-actin ratio (right) (*n* = 30 cells). Scale bar: 10 µm. *P < 0.05; **P < 0.01; ***P < 0.001.

Informed by RNA-sequencing analysis, we moved on to investigate the role of the PI3K/AKT signaling pathway in substrate stiffness-mediated regulation of microglial cell migration. Our results demonstrated that Pik3ca and AKT1 genes were upregulated in primary microglia on stiff substrates (Supplementary Figure S4A). Furthermore, the migration of BV2 cells on stiff substrates was significantly suppressed by treatment with LY294002 (LY) (Figure 6A and B), a commonly used and verified PI3K/AKT inhibitor (Supplementary Figure S4B). Furthermore, the levels of p-AKT and t-AKT protein expression were increased in BV2 cells cultured on stiff substrates, and these increases were downregulated following treatment with GsMTx4, although neither substrate stiffness nor GsMTx4 treatment affected the p-AKT/AKT ratio (Figure 6C and Supplementary Figure S5). Ca^2+^ chelation exerted a consistent effect on the t-AKT and p-AKT protein levels and the p-AKT/t-AKT ratio (Supplementary Figure S6). These findings indicate that AKT signaling is enhanced in response to Piezo1 channel activation and subsequently promotes cell migration on stiff substrates.

The PI3K/AKT signaling pathway is known to play a critical role in regulating actin cytoskeletal dynamics [57–60]. As introduced above, cofilin is an actin-binding protein that is activated by dephosphorylation and inactivated by phosphorylation, the latter of which may disrupt actin dynamics [38, 39, 41]. To investigate this mechanism, we focused on cofilin-mediated cytoskeletal reorganization. The level of cofilin phosphorylation was significantly reduced in BV2 cells cultured on stiff substrates compared to those on soft substrates. In addition, cofilin phosphorylation in BV2 cells on stiff substrates was increased by treatment with GsMTx4 (Figure 6D) or by LY-mediated inhibition of AKT signaling (Figure 6E). The total cofilin protein expression level remained unchanged under all tested conditions (Supplementary Figure S7). These findings suggest that Piezo1-mediated activation of AKT signaling promotes cofilin activation in BV2 cells cultured on stiff substrates. Subsequently, we examined the levels of F-actin and G-actin. Both F-actin and G-actin were elevated in BV2 cells on stiff substrates compared to those on soft substrates, but the G-actin to F-actin ratio was significantly reduced. The levels of F-actin and G-actin were decreased in BV2 cells on stiff substrates, and the G-actin to F-actin ratio was increased upon treatment with GsMTx4, BAPTA-AM or LY or following Piezo1 knockdown (Figure 6F–K and Supplementary Figures S8A and S9A and B). These results indicate that F-actin reassembly, which depends on G-actin polymerization, is facilitated by cofilin activation, and that cofilin functions as a downstream effector of the Piezo1/Ca^2+^/AKT signaling axis to enhance F-actin reassembly and microglial cell migration in response to stiff substrate stimulation.

### Piezo1/Ca^2+^ signaling-mediated regulation of AKT and cofilin activation is essential for F-actin disassembly/reassembly and stiff substrate-induced primary microglia migration

**Figure 7:**
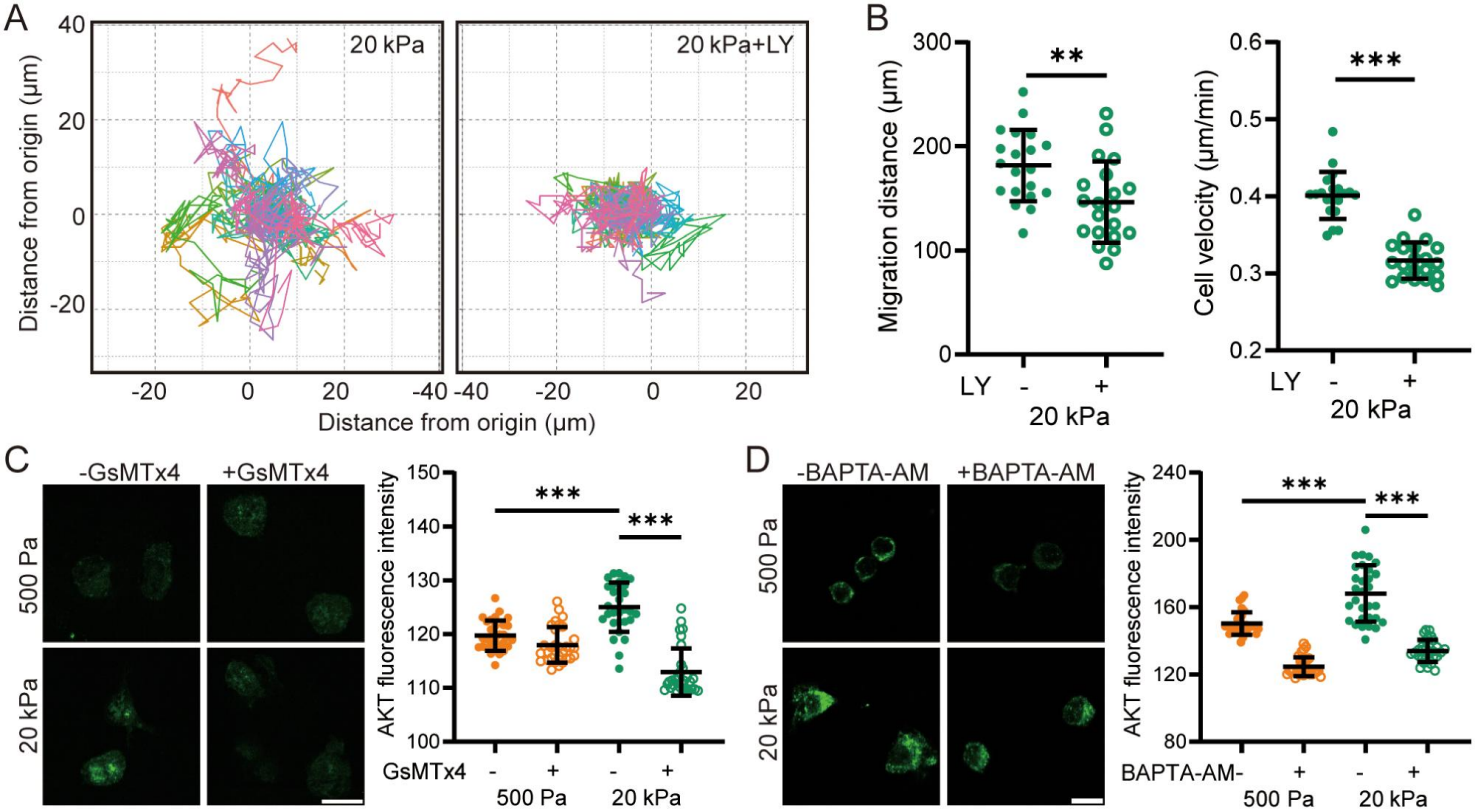
Activation of Piezo1-mediated and Ca^2+^-dependent AKT signaling pathway enhances migration of primary microglial cells on stiff substrates. (**A**, **B**) Migration analysis of cells seeded on 20 kPa substrates pretreated with DMSO or 20 μM LY for 1 h and subsequently tracked for 8 h (*n* = 20 cells). (**A**) Migration trajectories of individual cells; (**B**) Quantitative analysis of total migration distance (left) and average velocity (right). (**C**, **D**) Representative immunofluorescence images showing (left) and quantification (right) of AKT expression in cells seeded on 500 Pa and 20 kPa substrates. Cells were pretreated with 1 μM GsMTx4 (**C**) or 10 μM BAPTA-AM (**D**) for 1 h and subsequently cultured for a further 12 h (scale bar: 10 µm, *n* = 30 cells). **P < 0.01; ***P < 0.001.

**Figure 8:**
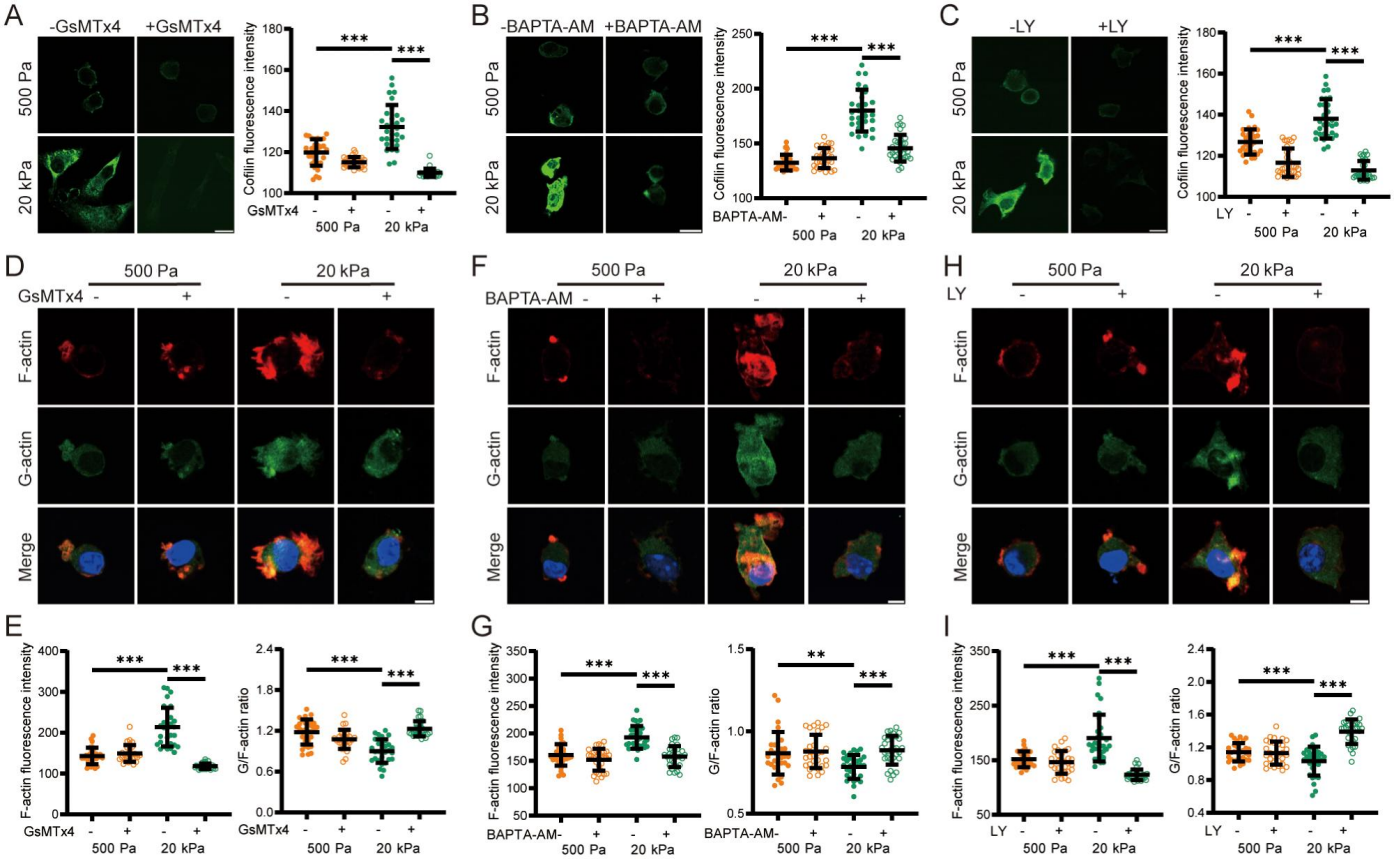
Piezo1/Ca^2+^/AKT Signaling-mediated regulation of cofilin activation and actin dynamics in primary microglial cells. (**A**–**C**) Representative immunofluorescence images showing (left) and quantification (right) of cofilin expression in cells seeded on 500 Pa and 20 kPa substrates. Cells were pretreated with 1 μM GsMTx4 (**A**) or 10 μM BAPTA-AM (**B**) or 20 μM LY (**C**) for 1 h and subsequently cultured for a further 12 h. Scale bar: 10 µm. (**D**, **E**) Representative immunofluorescence images showing expression of G-actin and F-actin (**D**) and quantification of F-actin protein expression (left of **E**) and G-actin/F-actin ratio (right of **E**) in cells on 500 Pa and 20 kPa substrates. Cells were pretreated with DMSO or 1 μM GsMTx4 for 1 h and subsequently cultured for a further 12 h. Scale bar: 5 µm. (**F**, **G**) Representative immunofluorescence images showing expression of G-actin and F-actin (**F**) and quantification of F-actin protein expression (left of G) and G-actin/F-actin ratio (right of G) in cells on 500 Pa and 20 kPa substrates. Cells were pretreated with DMSO or 10 μM BAPTA-AM for 1 h and subsequently cultured for a further 12 h. Scale bar: 5 µm (**H**, **I**). Representative immunofluorescence images showing expression of G-actin and F-actin (**H**) and quantification of F-actin protein expression (left of **I**) and G-actin/F-actin ratio (right of **I**) in cells on 500 Pa and 20 kPa substrates. Cells were pretreated with DMSO or 20 μM LY for 1 h and subsequently cultured for a further 12 h. Scale bar: 5 µm. *n* = 30 cells; **P < 0.01; ***P < 0.001.

We further investigated the role of the AKT signaling pathway in regulating substrate stiffness-mediated migration of primary microglial cells. Cell migration on stiff substrates was significantly reduced by treatment with LY (Figure 7A and B). Moreover, as shown by immunofluorescent staining, AKT protein expression was markedly higher in cells cultured on stiff substrates compared to those on soft substrates. This effect was suppressed by treatment with GsMTx4 (Figure 7C) or BAPTA-AM (Figure 7D). These findings demonstrate that the AKT signaling pathway acts as a downstream effector of the Piezo1 channel in the regulation of microglial cell migration by substrate stiffness. We finally examined cofilin protein expression and the dynamic changes in F-actin and G-actin in primary microglial cells. Cofilin protein expression was higher in cells cultured on stiff substrates compared to those on soft substrates. This increased expression was reduced by treatment with GsMTx4 (Figure 8A), BAPTA-AM (Figure 8B) or LY (Figure 8C). The levels of both F-actin and G-actin were elevated, and the G-actin to F-actin ratio was decreased in cells on stiff substrates relative to those on soft substrates. Furthermore, F-actin and G-actin levels were reduced, and the G-actin to F-actin ratio was increased in cells on stiff substrates following treatment with GsMTx4, BAPTA-AM or LY or Piezo1 knockdown (Figure 8D–I and Supplementary Figures S8B and S9C and D). These findings are consistent with results obtained in BV2 cells and provide further evidence that the Piezo1 channel plays a significant role in stiff substrate-induced microglial cell migration. Specifically, Piezo1-mediated elevation of [Ca^2+^]_i_ activates the AKT signaling pathway, promoting cofilin dephosphorylation and subsequent F-actin disassembly/reassembly to drive cell migration on stiff substrates.

## Discussion

The local stiffness of brain tissues under pathological conditions is significantly increased, which affects microglial cell migration. However, the underlying mechanism remains incompletely understood. In this study, we demonstrate the critical role of the mechanosensitive Piezo1 channel in determining migration of microglial cells on stiff substrates. Mechanistically, stiff substrates upregulate the Piezo1 channel expression and increase in [Ca^2+^]_i_, and Ca^2+^ subsequently enhances the AKT activity to promote cofilin dephosphorylation and facilitate F-actin reassembly by regulating actin dynamics, thereby driving cell migration.

### A positive role of the Piezo1 channel in stiff substrate-induced microglial cell migration

Migration is a pivotal ability of microglial cells to function under physiological conditions, particularly in pathological niches. For example, stiff amyloid-β plaques can activate the Piezo1 channel, thereby attracting microglial cells to migrate toward the plaques [22]. However, other studies suggest that stiff substrates upregulate the Piezo1 expression, and its silencing enhances cell migration on stiff substrates, as observed in BV2 cells cultured on soft and stiff substrates or primary microglial cells stimulated by amyloid-β [23]. Notably, cautions are required to interpret the results in these studies as cells were detached from the stiff environments prior to assessment of cell migration using the transwell assay. The present study utilized time-lapse video microscopy to examine cell migration *in situ* and revealed that microglial cell migration is enhanced on stiff substrates and is positively regulated by the Piezo1 channel (Figures 1 and 3), thereby reaffirming a positive role of the Piezo1 channel in mediating stiff substrate-induced microglial cell migration.

### The role of actin polymerization regulated by Piezo1-mediated rise [Ca^2+^]_i_ in stiff substrate-induced microglial cell migration

It is well established that the Piezo1 channel is critical in mediating microglial cell migration toward stiff amyloid-β plaques related to Alzheimer’s disease; however, the mechanisms by which Piezo1 regulates microglial cell migration remain unclear. Previous studies suggest that Piezo1 in microglial cells mediates stiff substrate-induced Ca^2+^ influx [22, 23]. Our results are consistent with these findings and further demonstrate that Piezo1-mediated Ca^2+^ influx is essential for enhanced microglial cell migration on stiff substrates (Figures 2A–D and 4A–D and Supplementary Figure S1). Additionally, microglial cells on stiff substrates exhibited actin cytoskeleton reorganization and polymerization, the processes that are required for cell migration. RNA-sequencing analysis of microglial cells isolated from 5×FAD mice with or without Piezo1 knockdown revealed that Piezo1 knockdown downregulates substrate-dependent cell migration signaling pathways, accompanied by decreases in Ca^2+^ signaling and actin cytoskeleton-related signaling, suggesting a potential but unverified linkage among these processes [22]. Here we provide evidence to show the critical role of Piezo1-mediated Ca^2+^ signaling in driving actin polymerization (F-actin reassembly) in stiff substrate-induced microglial cell migration (Figures 2 and 4). The critical regulatory role of the Piezo1 channel and related intracellular Ca^2+^ in cytoskeletal dynamics has been extensively documented in macrophages, indicating that Piezo1 acts as a central mediator of mechanical transduction, bridging mechanical signals from cell membrane to the cytoskeleton [49, 61].

### A critical role of AKT as a downstream effector of Piezo1-dependent Ca^2+^ signaling in stiff substrate-induced microglial cell migration

In this study, we have demonstrated that the AKT signaling pathway functions as a downstream effector of Piezo1-dependent Ca^2+^ signaling to regulate actin polymerization and microglial cell migration on stiff substrates (Figures 6A–C and 7). There is evidence that AKT activation regulates the phosphatase activity of Slingshot-1L, thereby promoting cofilin dephosphorylation and subsequent modulation of the actin cytoskeleton [62]. Previous studies have reported that both ERK1/2 and PI3K/AKT are activated in a Ca^2+^-dependent manner and regulate microglial cell migration in response to chemokine stimuli [63–65]. In our study, ERK1/2 is not involved, as its inhibition failed to affect microglial cell migration (Supplementary Figure S10), despite RNA-sequencing analysis suggesting substrate stiffness-dependent upregulation of the MAPK signaling (Figure 5B). Notably, stiff substrates increased the level of total AKT and p-AKT expression but not the ratio of p-AKT/AKT, which aligns with the findings from other studies [66, 67].

### The role of actin dynamics regulated by the Piezo1/Ca^2+^/AKT/cofilin signaling axis in stiff substrate-induced microglial cell migration

Previous studies indicate that actin reorganization and polymerization largely depend on the dephosphorylation or phosphorylation states of cofilin [38, 41]. It was also suggested that a higher level of p-cofilin inhibits the activity of cofilin to sever F-actin, thereby promoting actin polymerization and enhancing cell migration [68]. In contrast, other studies propose that cofilin-dependent F-actin severing is essential for actin polymerization as it increases the pool of G-actin, which facilitates actin polymerization or reassembly and promotes cell migration [38, 69]. Our results show that stiff substrates downregulated the level of p-cofilin (Figure 6D). Microglial cells migrated more rapidly on stiff substrates and exhibited frequent changes in cytoskeleton-mediated membrane protrusions (Supplementary Figure S11), with enhanced levels of G-actin (Supplementary Figure S8) and F-actin and reduced ratio of G-actin to F-actin (Figure 6F–K and 8D–I and Supplementary Figure S9). These results suggest that stiff substrates induce faster actin dynamics, including depolymerization/polymerization turnover, which enhances F-actin assembly and actin polymerization. Additionally, cofilin dephosphorylation was regulated by the Piezo1/Ca^2+^/AKT signaling axis, as the stiff substrate-induced decline in the level of p-cofilin was reversed upon inhibition of AKT or Piezo1 (Figure 6D and E). Previous studies have shown that the increase in [Ca^2+^]_i_ induces cofilin dephosphorylation via the action of protein phosphatases such as Slingshot [70]. Our current work extends the signaling pathway for cofilin dephosphorylation, suggesting that it is also regulated by Ca^2+^-dependent activation of AKT.

## Conclusion

In summary, our study has elucidated a detailed mechanism by which stiff substrates promote microglial cell migration through the mechanosensitive Piezo1 channel. Mechanistically, stiff substrates upregulate the Piezo1 expression and induce Piezo1-mediated extracellular Ca^2+^ influx to raise [Ca^2+^]_i_; Ca^2+^ in turn stimulates the AKT activity and cofilin dephosphorylation and promotes cofilin-dependent F-actin severing and subsequent actin polymerization, driving microglial cell migration on stiff substrates. Our findings provide a previously unrecognized mechanistic explanation, broadening our understanding of how stiff materials promote microglia migration, and offer insights for therapeutic strategies targeting brain pathologies associated with increased tissue stiffness.

## Supplementary Material

rbaf124_Supplementary_Data

## Data Availability

The data that support the findings of this study are available from the corresponding authors upon reasonable request.
