# Supplementary material for: Stiff matrix drives microglial cell migration through Piezo1/Ca2+/AKT/cofilin signaling axis-regulated F-actin reassembly
Source: Regen Biomater. 2026 Jan 12;13:rbaf124. doi: 10.1093/rb/rbaf124 (PMC12883088; doi:10.1093/rb/rbaf124)
Supplement: rbaf124_Supplementary_Data [file rbaf124_supplementary_data.pdf]

Stiff matrix drives microglial cell migration through Piezo1/ Ca^{2+}/AKT/cofilin signaling axis-regulated F-actin reassembly

Xinlan Chen[1†], Zhongchen Li[2†], Junqi Men[1], Hui Shao[1], Juncheng Bai[1], Yingying Guo[1], Xing Chen[1], Yubo Fan[1], Lin-Hua Jiang[3,4,5]*, and Xiaoling Jia[1]*

Table S1. **PA hydrogels formulations.**

Stiffness (kPa)	Acrylamide (%)	Bis-acrylamide (%)
0.5	3	0.06
1	5	0.03
20	8	0.264

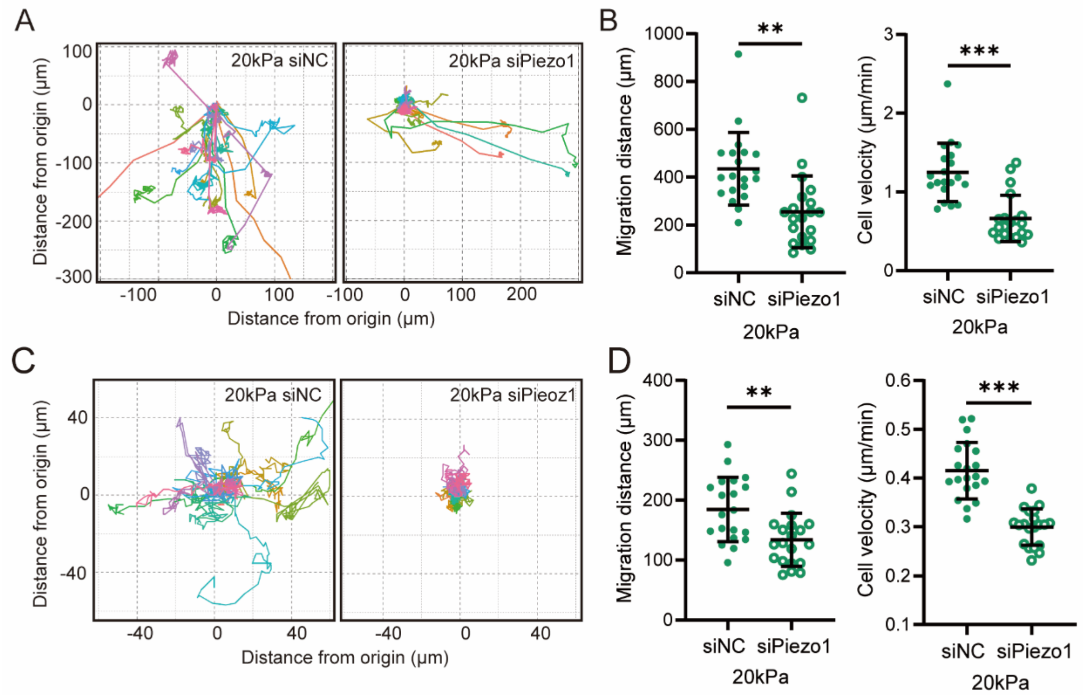

Figure S1. Piezo1 knockdown inhibited the migration of BV2 cells and primary microglial cells on stiff substrates. (A-B) Migration analysis of BV2 cells seeded on 20 kPa substrates

and transfected with negative control siRNA (siNC) or Piezo1-specific siRNA (siPiezo1). A: Migration trajectories of individual cells; B: Quantitative analysis of total migration distance and average velocity. (C-D) Migration analysis of primary microglial cells seeded on 20 kPa substrates and transfected with siNC or siPiezo1. A: Migration trajectories of individual cells; B: Quantitative analysis of total migration distance and average velocity. Statistics: mean ± SD; n = 20 cells; **P* < 0.05; ***P* < 0.01.

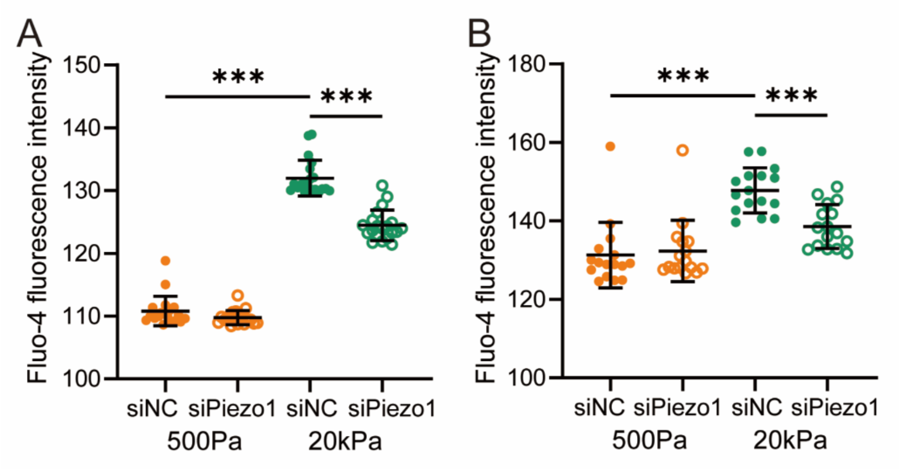

Figure S2. Piezo1 knockdown reduced the [Ca^{2+}]$_i$ of BV2 cells and primary microglial cells on stiff substrates. (A-B) Quantitative analysis of the average Fluo-4 fluorescence intensity in individual BV2 cells (A) and primary microglia cells (B). Cells were transfected with siNC or siPiezo1 on 500 Pa and 20 kPa substrates. Statistics: mean ± SD; n = 15 cells; ****P* < 0.001.

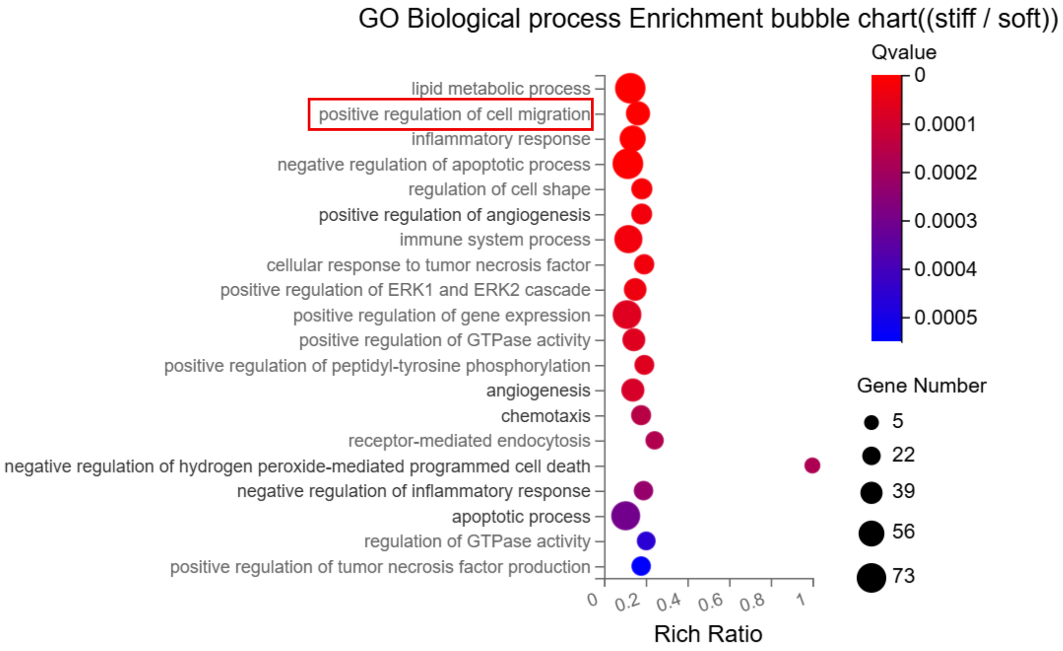

Figure S3. Enrichment analysis of DEGs in GO biological process category.

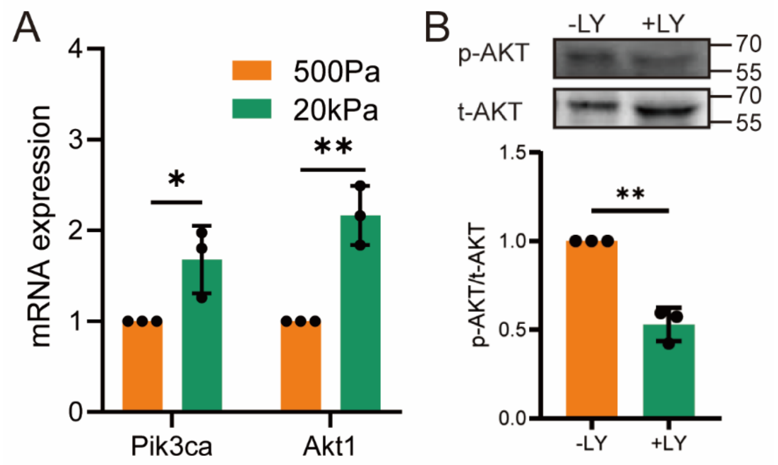

Figure S4. (A) Pik3ca and Akt1 mRNA expression in primary microglial cells cultured on 500 Pa and 20 kPa substrates. (B) LY treatment suppressed the phosphorylation of AKT. Statistics: mean ± SD; n = 3 repeats; *$P < 0.05$; **$P < 0.01$.

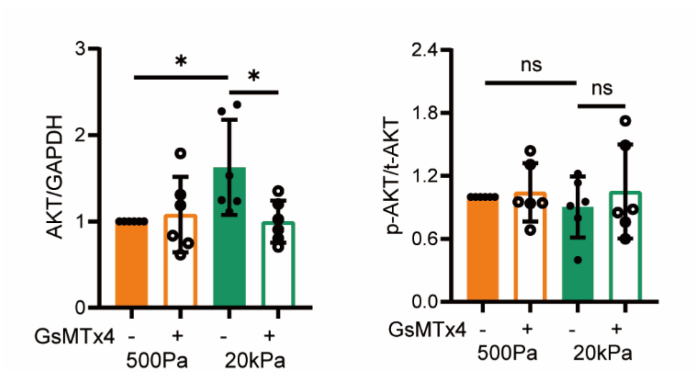

Figure S5. AKT protein expression in BV2 cells following GsMTx4 treatment. Quantification of the AKT/GAPDH and p-AKT/t-AKT in cells seeded on 500 Pa and 20 kPa substrates (n = 6 repeats). Cells were pretreated with DMSO or 1 μM GsMTx4 for 1 hour and subsequently cultured for further 12 hours.

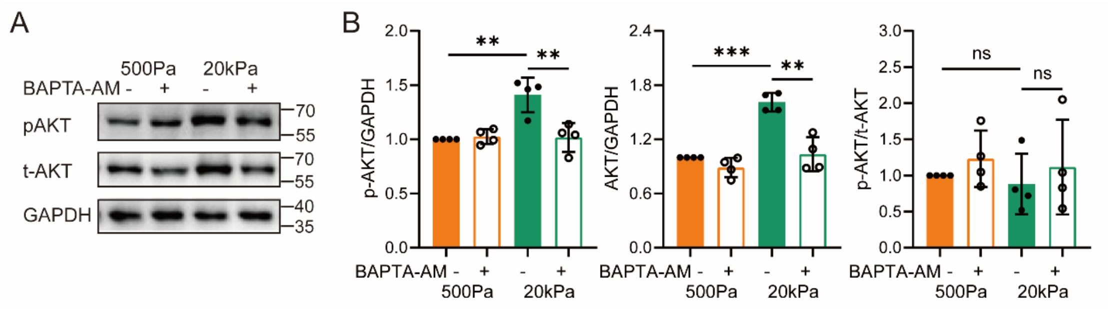

Figure S6. AKT protein expression in BV2 cells following BAPTA-AM treatment. Representative western blots showing (A) and quantification of AKT protein expression and the p-AKT/t-AKT ratio (B) in BV2 cells on 500 Pa and 20 kPa substrates (n = 4 repeats). Cells were pretreated with DMSO or 10 μM BAPTA-AM for 1 hour and subsequently cultured for a further 12 hours. Statistics: mean ± SD; ns, not significant; ***P* < 0.01; ****P* < 0.001; ns, not significant.

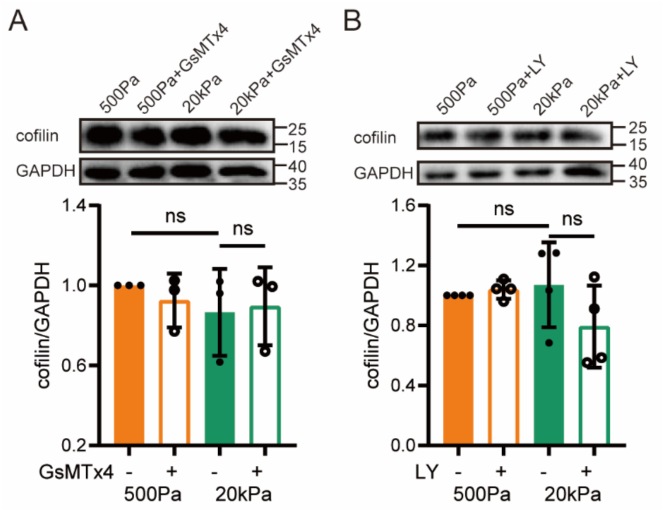

Figure S7. Cofilin protein expression in BV2 cells following GsMTx4 or LY treatment. (A) Representative western blots showing (top) and quantification of cofilin expression (bottom) in cells seeded on 500 Pa and 20 kPa substrates (n = 3 repeats). Cells were pretreated with DMSO or 1 μM GsMTx4 for 1 hour and subsequently cultured for further 12 hours. (D) Representative western blots showing (top) and quantification of cofilin expression (bottom) in cells seeded on 500 Pa and 20 kPa substrates (n = 4 repeats). Cells were pretreated with DMSO or 20 μM LY for 1 hour and subsequently cultured for further 12 hours. Statistics: mean ± SD; ns, not significant.

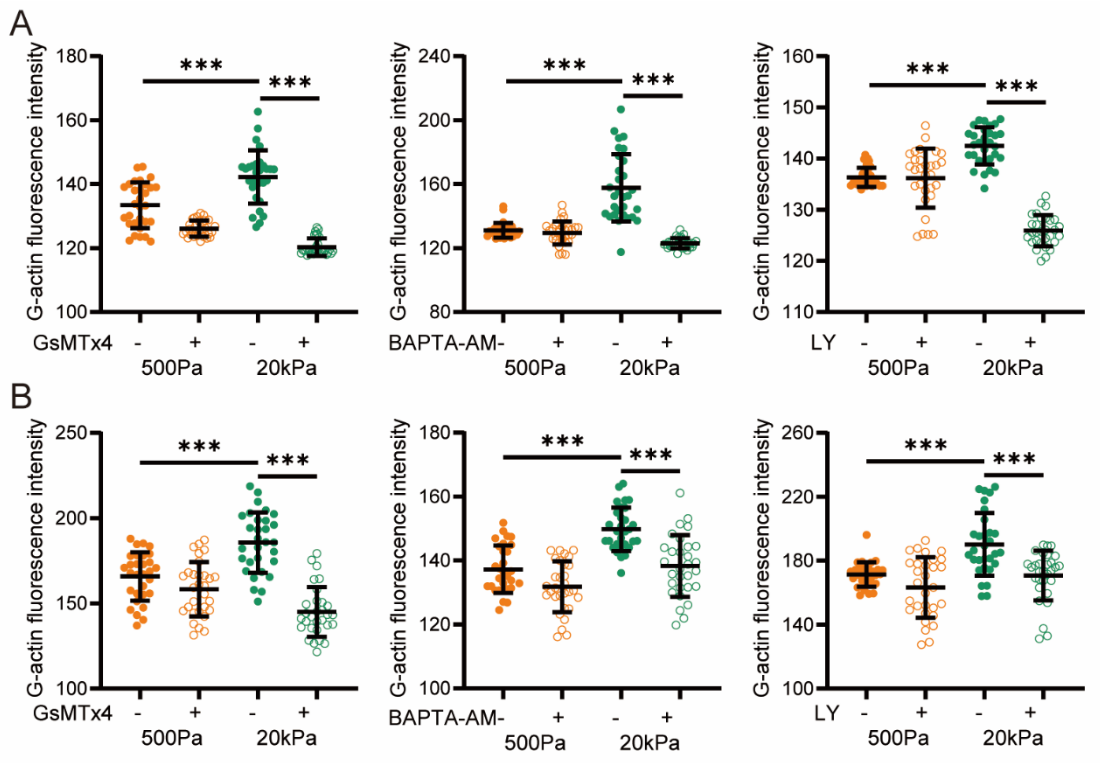

Figure S8. G-actin expression in BV2 cells and primary microglial cells. (A) Quantification of G-actin expression in BV2 cells growing on soft or stiff substrates and treated with GsMTx4, BAPTA-AM, or LY. Representative images are presented in Figures 6F, H, J. (B) Quantification of G-actin expression following treatment with GsMTx4, BAPTA-AM, or LY on soft or stiff substrates in primary microglial cells. Representative images are presented in Figures 8D, F, H. Statistics: mean ± SD; n = 30 cells; ****P* < 0.001.

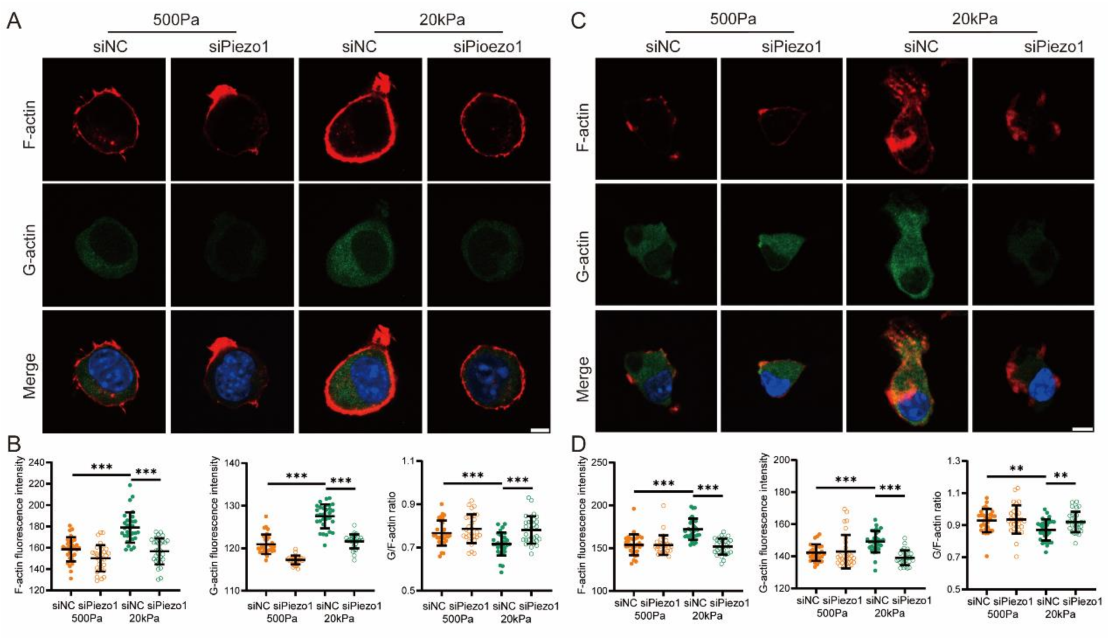

Figure S9 Piezo1 knockdown regulated the actin dynamics in BV2 cells and primary microglial cells. (A-B) Representative immunofluorescence images showing expression of G-actin and F-actin (A) and quantification of F-actin, G-actin protein expression, and G-actin/F-actin ratio (B) in BV2 cells. Cells were transfected with siNC or siPiezo1 on 500 Pa and 20 kPa substrates. (C-D) Representative immunofluorescence images showing expression of G-actin and F-actin (C) and quantification of F-actin, G-actin protein expression, and G-actin/F-actin ratio (D) in primary microglial cells. Cells were transfected with siNC or siPiezo1 on 500 Pa and 20 kPa substrates. Statistics: mean ± SD; n = 30 cells; ***$P < 0.001$.

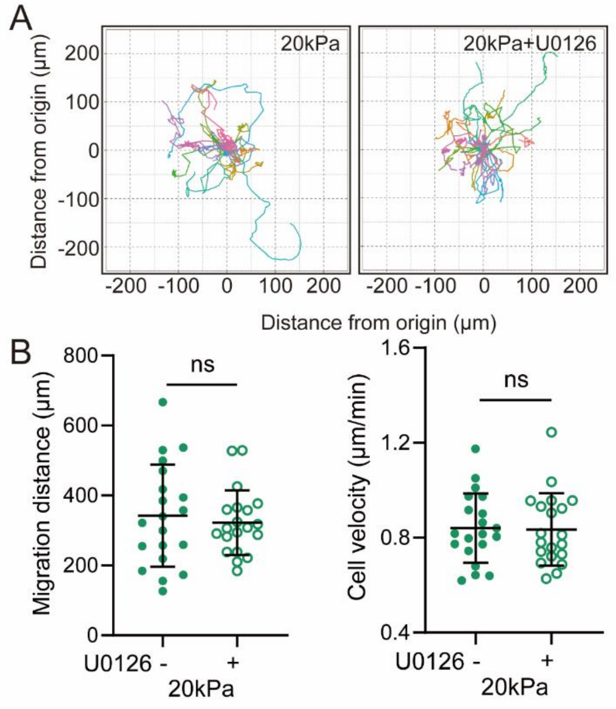

Figure S10. Blockade of the ERK1/2 signaling pathway does not affect migration of BV2 cells. (A) Migration trajectories of individual cells seeded on 20 kPa substrates pretreated with DMSO or 10 μM U0126 for 1 hour and subsequently tracked for 8 hours. (B) Quantitative analysis of total migration distance (left) and average velocity (right) in panel A. Statistics: mean ± SD; n = 20 cells; ns, not significant.

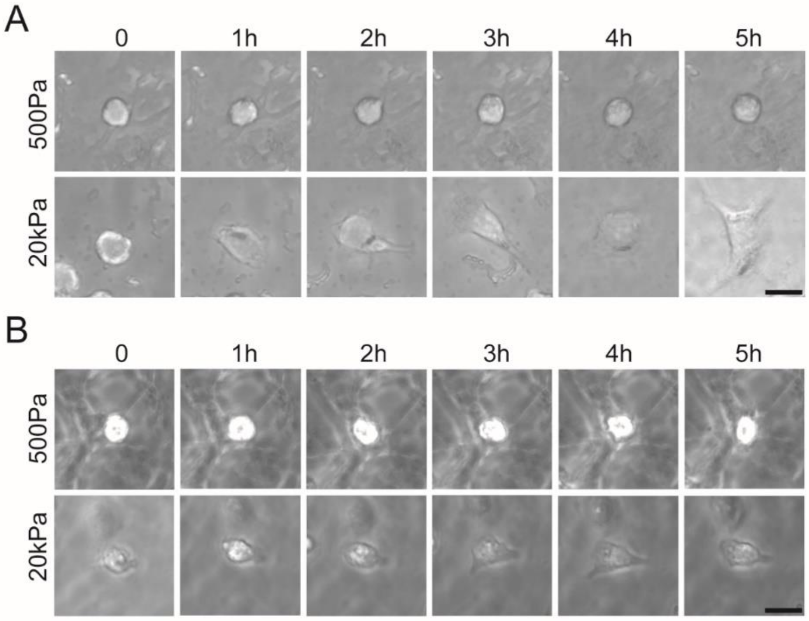

Figure S11. Time-dependent morphological changes of microglial cells on substrates with different stiffness during migration. (A) Bright-field microscope images of BV2 cells on 500 Pa or 20 kPa substrates during the 5-hour cell migration process, displayed at 1-hour intervals. (B) Bright-field microscope images of primary microglial cells on 500 Pa or 20 kPa substrates during the 5-hour cell migration process, displayed at 1-hour intervals. Scale bar: 20 μm.
